# O-GlcNAcylation promotes colorectal cancer metastasis via the miR-101-O-GlcNAc/EZH2 regulatory feedback circuit

**DOI:** 10.1038/s41388-018-0435-5

**Published:** 2018-08-09

**Authors:** Mingzuo Jiang, Bing Xu, Xiaowei Li, Yulong Shang, Yi Chu, Weijie Wang, Di Chen, Nan Wu, Sijun Hu, Song Zhang, Mengbin Li, Kaichun Wu, Xiaoyong Yang, Jie Liang, Yongzhan Nie, Daiming Fan

**Affiliations:** 1 State key Laboratory of Cancer Biology, National Clinical Research Center for Digestive Diseases and Xijing Hospital of Digestive Diseases, Fourth Military Medical University, Xi’an, China; 2 Department of Gastroenterology, Second Affiliated Hospital of Xi’an Jiaotong University, Xi’an, 710004 Shaanxi Province China; 3 Lab of Tissue Engineering, Faculty of Life Science, Northwest University, Xi’an, China; 4 Department of molecular cellular and developmental biology, Yale University, New Haven, USA

**Keywords:** Colorectal cancer, Metastasis

## Abstract

Advanced colorectal cancer (CRC) is one of the deadliest cancers, and the 5-year survival rate of patients with metastasis is extremely low. The epithelial–mesenchymal transition (EMT) is considered essential for metastatic CRC, but the fundamental molecular basis underlying this effect remains unknown. Here, we identified that O-GlcNAcylation, a unique posttranslational modification (PTM) involved in cancer metabolic reprogramming, increased the metastatic capability of CRC. The levels of O-GlcNAcylation were increased in the metastatic CRC tissues and cell lines, which likely promoted the EMT by enhancing EZH2 protein stability and function. The CRC patients with higher levels of O-GlcNAcylation exhibited greater lymph node metastasis potential and lower overall survival. Bioinformatic analysis and luciferase reporter assays revealed that both O-GlcNAcylation transferase (OGT) and EZH2 are posttranscriptionally inhibited by microRNA-101. In addition, O-GlcNAcylation and H3K27me3 modification in the miR-101 promoter region further inhibited the transcription of miR-101, resulting in the upregulation of OGT and EZH2 in metastatic CRC, thus forming a vicious cycle. In this study, we demonstrated that O-GlcNAcylation, which is negatively regulated by microRNA-101, likely promotes CRC metastasis by enhancing EZH2 protein stability and function. Reducing O-GlcNAcylation may be a potential therapeutic strategy for metastatic CRC.

## Introduction

Tumor metastasis represents a multistep cellular biological event termed the invasion-metastasis cascade, whereby epithelial cells in primary tumors disseminate as cancer cells to anatomically distant organs and subsequently adapt to the foreign microenvironments [1]. Increased risk of cancer-related death is a serious consequence of metastasis [2]. Despite significant advances in the detection and treatment of colorectal cancer (CRC), many patients still die from local or distant metastasis [2–4]. Although a substantial number of molecules have been identified that reveal important aspects of CRC metastasis, the critical molecular basis behind CRC metastasis is still largely unknown. In addition, most of the current investigations are focused on genetic mechanisms and cellular signaling pathways, and little is known about the metabolic and epigenetic mechanisms involved in this process.

Cancer cells reprogram their metabolism to shift away from oxidative phosphorylation and toward anaerobic glycolysis, termed the Warburg effect, even in the presence of adequate oxygen, leading to significant increase in glucose uptake [5]. A high rate of aerobic glycolysis and glucose uptake upregulates the hexosamine biosynthetic pathway (HBP) flux, ultimately leading to an increase in UDP-β-d-N-acetylglucosamine (UDP-GlcNAc) levels, the end product of the HBP [6–8]. Then, UDP-GlcNAc is attached to serine or threonine residues on intracellular proteins, catalyzed by a unique enzyme known as O-GlcNAcylation transferase (OGT) and removed by O-GlcNAcase (OGA) [9, 10]. Most studies confirmed that O-GlcNAcylation is generally elevated in a variety of cancers owing to the Warburg effect, and it has been recognized as a lynchpin for the development and progression of many types of malignancies [9, 11–13]. O-GlcNAcylation has been identified as a dynamic and reversible posttranslational modification that regulates diverse cellular processes, such as cell signal transduction, protein translation and proteasomal degradation [14–18]. Recently, O-GlcNAcylation was found to be increased in CRC cells to enable the proliferative and migratory properties of these cells [19]. However, the potential effect of O-GlcNAcylation and the mechanism of its action in CRC metastasis are still not well understood.

In addition to O-GlcNAcylation, OGT, which is the enzyme that catalyzes O-GlcNAcylation, is also increased in various cancers [9, 11, 20, 21], indicating that other mechanisms in addition to metabolism regulate O-GlcNAcylation. Indeed, evidence suggests that the mTOR signaling pathway regulates OGT and O-GlcNAcylation expression in breast [22] and colon [23] cancer. In addition to transcriptional regulation, posttranscriptional regulation is crucial in the regulation of OGT and O-GlcNAcylation. It has been reported that microRNAs (miRNAs), small noncoding RNA molecules, are involved in the regulation of OGT expression in both normal and malignant cells. Recent studies have found that miR-7 [24] and miR-423-5p [25] directly regulate OGT mRNA decay in endothelial cells and cardiomyocytes, respectively. Notably, a recent study has found that miR-485 reduces the O-GlcNAcylation of Bmi-1 and inhibits CRC proliferation by directly regulating OGT [26]. Recently, the development of network databases has provided a powerful tool for the prediction of miRNA targets, and it has been well documented that miRNAs play an important role in CRC [27–29]. As both miRNAs and O-GlcNAcylation may play important roles in CRC metastasis, we are interested in determining whether O-GlcNAcylation is regulated by certain miRNAs to further regulate metastasis in CRC.

In this study, we identified that O-GlcNAcylation is a central component linking metabolism and epigenetic regulation to CRC metastasis through miR-101 and EZH2. The downregulation of miR-101 in CRC promotes the elevation of O-GlcNAcylation and, thus, enhances EZH2 protein stability and function, which, in turn, further reduces the expression of miR-101. In addition, reducing O-GlcNAcylation may be a potential therapeutic strategy for metastatic CRC, providing new insight into the treatment of metastatic CRC in humans.

## Results

### O-GlcNAcylation is significantly upregulated in human CRC tissues and positively associated with lymph node metastasis

**Fig. 1:**
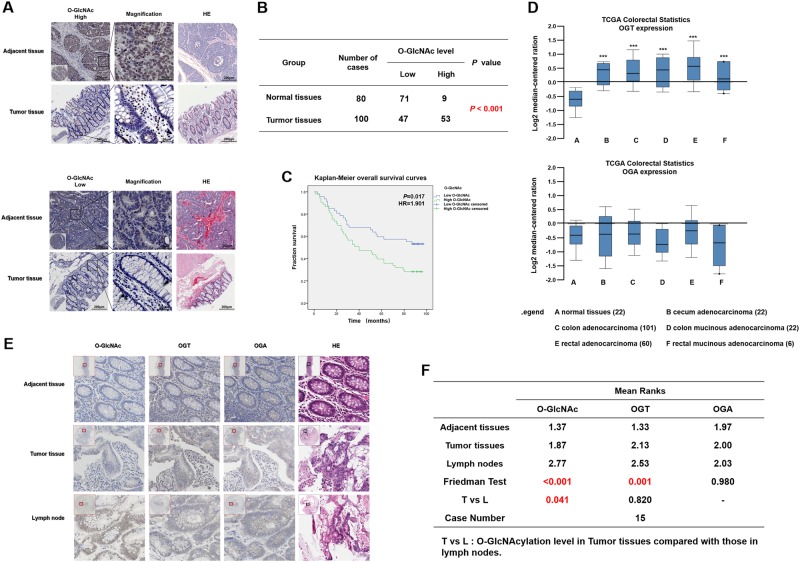
O-GlcNAcylation is significantly upregulated in human colorectal cancer tissues. **a** Representative images of immunohistochemical (IHC) staining of O-GlcNAcylation and hematoxylin-eosin (HE) staining in 100 CRC tissues and 80 adjacent normal tissues. The high (upper) and low (lower) expression levels of O-GlcNAcylation were evaluated semiquantitatively by the staining intensity (high score: 8–12; low score: 0–6). **b** Chi-square analysis of the O-GlcNAcylation level in 100 CRC tissues and 80 adjacent normal tissues. **c** Kaplan–Meier curve depicting the overall survival of CRC patients (*n* = 100). The curves were stratified based on the O-GlcNAcylation level. Overall survival was defined as the interval between the date of surgery and the date of death or last follow-up. **d** OGT and OGA mRNA levels in 211 CRC tumor samples and 22 normal controls from the Cancer Genome Atlas (TCGA) data. The data were analyzed by ONCOMINE. **e** Representative images of IHC staining of O-GlcNAcylation and HE staining in adjacent normal tissues, tumor tissues, and positive metastatic lymph nodes. **f** Friedman rank-sum test of O-GlcNAcylation level in 15 cases of adjacent normal tissues, tumor tissues, and positive metastatic lymph nodes. T *vs*. L refers to the O-GlcNAcylation level in CRC tissues compared with that in the positive metastatic lymph nodes

To investigate the expression of O-GlcNAcylation in CRC tissues, CRC tissue microarrays were used. The immunohistochemical (IHC) staining of the CRC tissue microarrays showed that O-GlcNAcylation was predominantly localized to both the cytoplasm and nucleus and significantly elevated in cancer tissues compared with that in normal tissues (57.78% vs 11.25%, *P* *<* 0.01) (Fig. 1a, b). High O-GlcNAcylation was not correlated with age, gender, tumor location, or size (*P* *>* 0.05, Table 1) but was significantly positively associated with lymph node metastasis and the American Joint Committee on Cancer (AJCC) status (*P* < 0.01, Table 1). In addition, the patients with higher O-GlcNAcylation had shorter overall survival than those with lower O-GlcNAcylation, indicating that the O-GlcNAcylation level was an independent risk factor for a poor prognosis in CRC patients (Fig. 1c, Tables 2 and 3, *P* *<* 0.05)*.*

**Table 1:** Prognostic factors in colon cancer patients by univariate analysis

Parameter	*n*	Cumulative survival rates (%) 3-Years 5-Years		Mean survival time (mo)	Hazard ratio	95% Confidence interval	*P* value
Gender							
Male	55	61.8	49.1	58.5	0.99	0.60–1.65	0.967
Female	45	55.6	48.9	57.1			
Age							
< 60	26	69.2	55.4	68.4	0.53	0.27–1.02	0.057
≥ 60	74	55.4	44.6	54.0			
Location							
Colon ascendens	49	53.1	46.9	52.9			0.137
Colon transversum	11	45.5	27.3	42.6	1.88	0.99–3.57	0.055
Colon descendens	12	58.3	50.0	60.8	2.44	1.01–5.91	0.047
Colon sigmoideum/rectum	28	75	60.7	70.8	1.22	0.46–3.22	0.684
Tumor size							
< 5 cm	39	71.8	51.3	63.4	0.78	0.46–1.32	0.362
≥ 5 cm	61	50.8	47.5	54.4			
Lymph node metastasis							
Negative	62	74.2	61.3	68.3	0.41	0.25–0.69	< 0.001
Positive	38	34.2	28.9	40.2			
AJCC stage							
I	8	62.5	62.5	62.8			< 0.001
II	54	75.9	61.4	68.7	0.06	0.01–0.27	< 0.001
III	35	37.1	31.4	43.0	0.05	0.01–0.19	< 0.001
IV	3	0	0	8.0	0.11	0.03–0.42	< 0.001
O-GlcNAcylation level							
Low	47	68.1	59.6	66.8	0.53	0.31–0.89	0.017
High	53	50.9	39.6	49.7			
Overall	100	59.0	49.0	57.9

**Table 2:** Association of O-GlcNAcylation level with clinicopathological parameters of patients with colon cancer

Parameter	*n*	O-GlcNAcylation level		*P* value
		Low	High	
Gender				
Male	55	30	25	0.095
Female	45	17	28	
Age				
< 60	26	14	12	0.416
≥ 60	74	33	41	
Location				
Colon ascendens	49	22	27	0.773
Colon transversum	11	4	7	
Colon descendens	12	6	6	
Colon sigmoideum/rectum	28	15	13	
Tumor size				
< 5 cm	39	22	17	0.132
≥ 5 cm	61	25	36	
Lymph node metastasis				
Negative	62	36	26	0.005
Positive	38	11	27	
AJCC stage				
I	8	6	2	0.028
II	54	30	24	
III	35	10	25	
IV	3	1	2

**Table 3:** Multivariate analysis using the Cox proportional hazards model

Parameter	*n*	Hazard ratio	95% confidence interval	*P* value
Gender				
Male	55	0.741	0.427–1.285	0.285
Female	45			
Age				
< 60	26	0.663	0.340–1.293	0.228
≥ 60	74			
Tumor size				
< 5 cm	39	0.750	0.433–1.300	0.306
≥ 5 cm	61			
Lymph node metastasis				
Negative	62	0.416	0.234–0.739	0.003
Positive	38			
O-GlcNAcylation level				
Low	47	0.650	0.513–0.932	0.041
High	53			

O-GlcNAcylation is well known to be dynamically regulated by OGT and OGA [30]. To investigate the expression of OGT and OGA in human CRC tissues, we reviewed the Cancer Genome Atlas (TCGA) by the Oncomine database and found that the OGT mRNA level was significantly elevated in all types of CRC, including cecum adenocarcinoma, colon adenocarcinoma, colon mucinous adenocarcinoma, rectal adenocarcinoma, and rectal mucinous adenocarcinoma, compared with that in normal colorectal tissues (Fig. 1d). However, there was no significant change in the mRNA level of OGA in CRC (Fig. 1d). This finding suggests that the increase in the O-GlcNAcylation levels in CRC may be mainly due to the increase in OGT expression and that the O-GlcNAcylation level is correlated with the metastatic capacity of CRC cells.

To further verify the relationship between O-GlcNAcylation and the metastasis of CRC, we selected 15 cases of CRC patients with lymph node metastases and detected the expression of O-GlcNAcylation, OGT and OGA in the adjacent tissues, tumor tissues, and lymph node metastases, respectively (Fig. 1e). As shown in Fig. 1f, the expression of O-GlcNAcylation and OGT significantly differed among the adjacent tissues, tumor tissues, and lymph node metastases. However, the expression of O-GlcNAcylation only significantly differed between the tumor tissues and lymph node metastases (Fig. 1f). Therefore, we further studied the mechanism by which O-GlcNAcylation promotes metastasis in CRC.

### O-GlcNAcylation promotes migratory and invasive capacities of CRC cells in vitro and in vivo

**Fig. 2:**
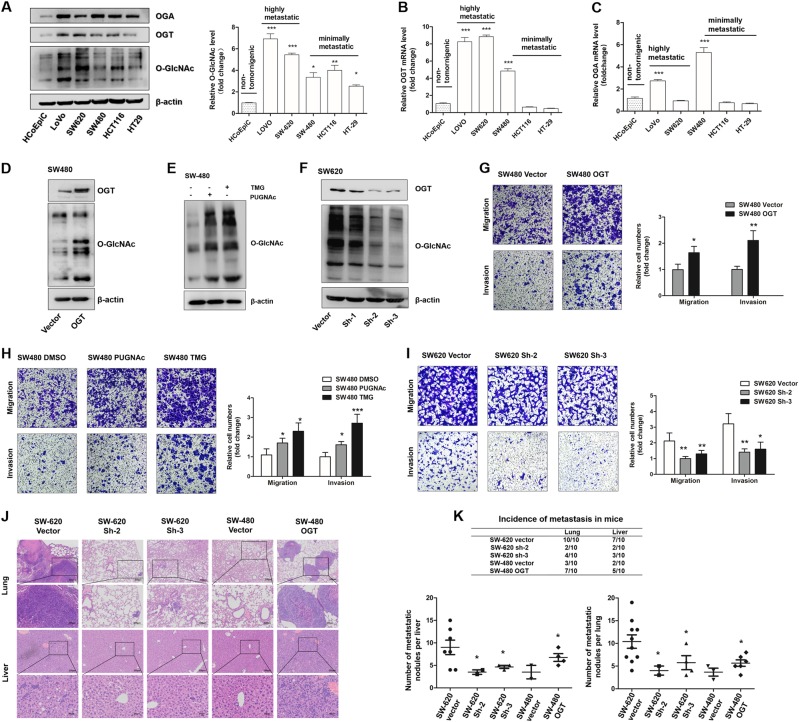
O-GlcNAcylation promotes migratory and invasive capacities of colorectal cancer cells. **a** Western blotting of OGA, OGT, and O-GlcNAcylation in five human CRC cell lines and one normal human intestinal epithelial cell line. The mean level of O-GlcNAcylation from three independent biological replicates is shown on the right. The values shown are expressed as the means ± SEM. * represents Student’s *t* test *P < 0.05, **P < 0.01, and ***P < 0.001. **b**, **c** qRT-PCR of OGT and OGA in five human CRC cell lines and one normal human intestinal epithelial cell line. The values shown are expressed as the means ± SEM. *** represents Student’s *t* test *P* < 0.001. **d** SW480 cells were transfected with the LV-OGT expression vector or vector control. Western blotting of OGT and O-GlcNAcylation. **e** Western blotting of O-GlcNAcylation in SW480 cells after treatment with PUGNAc (100 mol/L), Thiamet-G (TMG, 10 μmol/L), or isometric DMSO (negative control, NC) for 12 h**. f** SW620 cells were transfected with LV-OGT-shRNA (Sh-1, Sh-2, and Sh-3) or the control vector. The OGT expression and O-GlcNAcylation levels were determined by western blotting. **g**–**i** Transwell assay of SW480 or SW620 cells with the indicated treatment. * represents Student’s *t* test *P < 0.05, **P < 0.01, and ***P < 0.001. **j** SW620 (Vector, Sh-2, and Sh-3 groups) and SW480 (Vector and OGT groups) cells were injected into nude mice via the tail vein, and the animals were killed 7 weeks after the injections. Representative HE staining of lung (up) or liver (down) tissue samples are shown. **k** The incidence of lung and liver metastasis in each group of nude mice (up); the number of liver or lung metastatic foci observed in each group (bottom). *represents Kruskal–Wallis test *P* < 0.05

To further confirm our hypothesis, we examined the expression of OGT, OGA, and O-GlcNAcylation in five human CRC cell lines (LoVo, SW620, SW480, HCT-116, and HT-29) and the normal human intestinal epithelial cell line HCoEpiC. Although the expression of OGT and OGA in the various cell lines was not uniformly increased or decreased, the O-GlcNAcylation level was significantly increased in all cancer cell lines compared with that in the HCoEpiC cell line (Fig. 2a–c). Importantly, the cell lines with a high metastatic potential, i.e., LoVo and SW620, which were established from distant metastatic foci [31], exhibited higher expression levels of O-GlcNAcylation than the other CRC cell lines (Fig. 2a). As the SW620 and SW480 cell lines had a similar genetic background but different metastatic propensities [32], we selected these cell lines for further investigation. Because O-GlcNAcylation is only dynamically regulated by OGT and OGA [30], we interfered with the basal level of O-GlcNAcylation by manipulating the OGT gene or inhibiting OGA activity using potent inhibitors (Fig. 2d–f). The transwell assays showed that the migration and invasion capacities were markedly increased (Fig. 2g, h) when the O-GlcNAcylation level was upregulated by the overexpression of OGT (Fig. 2d) or treatment with the O-GlcNAcylationase inhibitors PUGNAc and Thiamet-G (Fig. 2e). However, in the SW620 cells, these capacities were decreased (Fig. 2i) when OGT was knocked down. (Fig. 2f).

To better simulate the in vivo environment, we further evaluated the effect of O-GlcNAcylation on metastatic capability through 3D spheroid basal membrane extract (BME) cell invasion assays [33]. Indeed, cancer cell spheroids with higher O-GlcNAcylation levels had larger invasion areas (Supplemental Fig. 1A and B). Meanwhile, the xenograft model also revealed that the number of metastatic nodules in the liver and lung was dramatically decreased when O-GlcNAcylation was downregulated in the SW620 cells but increased when OGT and O-GlcNAcylation were upregulated in the SW480 cells (Fig. 2j, k), indicating that O-GlcNAcylation promotes the metastatic capacity of colon cancer cells in vivo. In summary, these results strongly indicated that O-GlcNAcylation promotes the migratory and invasive capacities of colon cancer cells in vitro and in vivo.

### O-GlcNAcylation may contribute to metastasis in CRC by regulating the EMT via EZH2

**Fig. 3:**
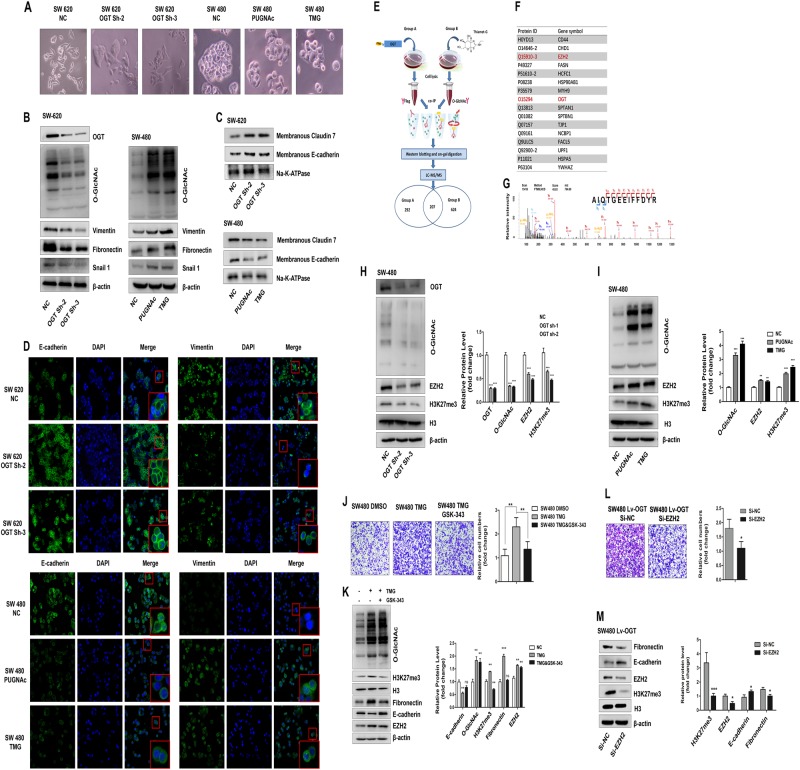
O-GlcNAcylation promotes colorectal cancer metastasis by regulating the EMT via EZH2. **a** Morphological changes by O-GlcNAcylation in SW620 and SW480 cells. Photographs using the × 20 objective. **b**–**d** Western blotting and immunofluorescence staining reveal an increased localization of epithelial markers in the membrane and downregulated expression of mesenchymal markers in SW620-Sh-1 and SW620-Sh-2 cells. In contrast, the upregulation of O-GlcNAcylation after 12 h of treatment with PUGNAc (100 mol/L) or Thiamet-G (10 μmol/L) resulted in a decreased localization of epithelial markers in the membrane and increased expression of mesenchymal markers in SW480 cells. **e** Workflow used for the identification of putative O-GlcNAc-modified proteins in SW480 cells. **f** A representative list of putative O-GlcNAc-modified proteins. The protein IDs from UniProt and gene symbols are shown. **g** The MS/MS spectrum and sequencing results of an unmodified peptide from in-gel digestion. The peptide was identified as AIQTGEEIFFDYR, corresponding to the 715–727 fragment of EZH2. **h**, **i** Western blot showing the levels of EZH2 protein and H3K27me3 in SW480 cells transfected with ShRNA-OGT (Sh-2 and Sh-3) or the control vector (**h**) or SW480 cells after a 12-hour treatment with PUGNAc (100 mol/L), Thiamet-G (TMG, 10 μmol/L), or isometric DMSO (negative control, NC) (**i).** The mean level of the indicated protein from three independent biological replicates is shown on the right. The values shown are expressed as the means ± SEM. ** represents Student’s *t* test *P* < 0.01 and ***P < 0.001. **j**, **k** Transwell assay and western blot analysis of the indicated proteins in SW480 cells after a 12-hour treatment with TMG (10 μmol/L) alone or in combination with GSK-343 (3 μmol/L) for 3 days. Isometric DMSO treatment was used as a negative control. **l**, **m** Transwell assay and western blot analysis of the indicated proteins in SW480 Lv-OGT cells transfected with EZH2 siRNA or negative control

We found that the knockdown of OGT in SW620 resulted in a morphological transition from a fibroblast-like morphology to a tighter organization of cells in colonies. In contrast, increasing the O-GlcNAcylation level in SW480 cells promoted the reverse process (Fig. 3a). This phenomenon suggested that O-GlcNAcylation may promote CRC metastasis by regulating the EMT. Indeed, the expression of fibronectin and vimentin, which are the major markers of mesenchymal cells, decreased when OGT was downregulated in the SW620 cells (Fig. 3b), whereas the localization of Claudin 7 and E-cadherin on the cell membrane (membranous Claudin 7 and E-cadherin) was increased (Fig. 3c). Consistent with these results, these markers showed the opposite change when O-GlcNAcylation was upregulated by PUGNAc or TMG treatment in the SW480 cells (Fig. 3b, c). In addition, the expression of snail1, which is a key transcription factor in the induction of the EMT, consistently changed with the level of O-GlcNAcylation (Fig. 3b). These changes in the expression of E-cadherin and vimentin were confirmed by an immunofluorescent assay (Fig. 3d).

To elucidate the exact mechanism by which O-GlcNAcylation promotes metastasis in CRC, a co-immunoprecipitation (Co-IP) assay was performed in SW480 OGT overexpression cells using OGT and O-GlcNAc antibodies (Fig. 3e). In the O-GlcNAc Co-IP group, the cells were treated with Thiamet-G 48 h prior to performing the assay (Fig. 3e). The Co-IP proteins were analyzed by LC-MS/MS to search for putative O-GlcNAcylation targets that were catalyzed by OGT, and ~ 200 proteins were observed in both groups simultaneously (Fig. 3e, f, Table S1). Among them, histone methyltransferase enhancer of zeste homolog 2 (EZH2) (Fig. 3g), the enzyme that catalyzes the tri-methylation of lysine 27 on histone H3 (H3K27me3), is a known, key epigenetic regulator and EMT inducer, which participates in the metastasis of a variety of cancers [34–37].

Then, we investigated whether O-GlcNAcylation promotes the EMT via EZH2 in CRC cells. We observed that the levels of EZH2 protein and H3K27me3 were significantly reduced when O-GlcNAcylation was downregulated in the SW480 cells (Fig. 3h), whereas these levels were increased after PUGNAc or TMG treatment (Fig. 3i). Meanwhile, the 3D spheroid BME cell invasion assays (Supplemental Fig. 1C), transwell migration assays (Fig. 3j) and western blotting assays (Fig. 3k) showed that GSK-343, which is a specific inhibitor of EZH2 (Supplemental Fig. 2D), significantly blocked the upregulated invasiveness and EMT effect induced by hyper-O-GlcNAcylation. In addition, the migration abilities of the SW480 LV-OGT cells were significantly decreased following the knockdown of EZH2 (Fig. 3l), and the protein level of Fibronectin in the SW480 LV-OGT cells was substantially decreased when EZH2 was downregulated, but E-cadherin was increased (Fig. 3m). These data indicated that EZH2 plays an important role in promoting the migration and invasion of CRC by O-GlcNAcylation, and the knockdown of EZH2 could partially reverse the O-GlcNAcylation-mediated EMT.

### O-GlcNAcylation modification enhances EZH2 protein stability and function

**Fig. 4:**
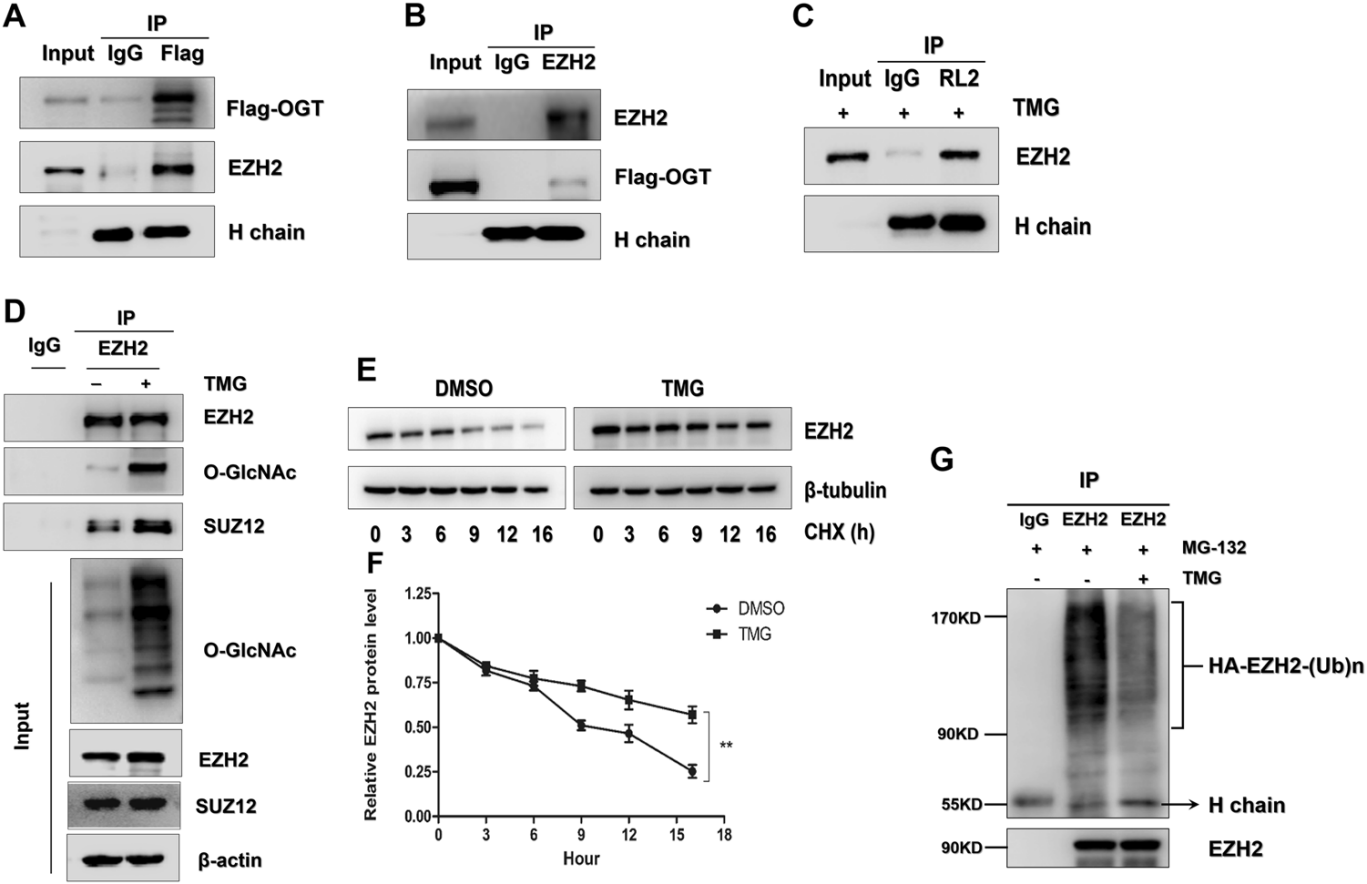
O-GlcNAcylation promotes EZH2 protein stability and function. **a**, **b** Total lysates from SW480 cells expressing OGT-FLAG were subjected to IP with FLAG Ab or EZH2 Ab, followed by western blotting using the indicated antibodies (Abs). Ig heavy chain (H Chain) was used as a loading control. **c** Total lysates from SW480 cells treated with TMG were subjected to IP with IgG or O-GlcNAcylation Ab (RL2), followed by western blotting using EZH2 Ab. Heavy Chain (H Chain) was used as a loading control. **d** Total lysates from SW480 cells with or without TMG were subjected to IP with IgG or EZH2, followed by western blotting using O-GlcNAcylation, EZH2, or SUZ12 Ab. **e**, **f** The half-life of EZH2 in the presence/absence of TMG. Cells were treated with cycloheximide (CHX) for the indicated times; then, EZH2 level was analyzed by western blotting. β-Tubulin was used as a loading control. Normalization was completed by dividing the EZH2 signal by the β-tubulin signal. **g** Western blot analysis of ubiquitin on EZH2 immunoprecipitated from SW480 cells stably expressing FLAG-EZH2 in the presence or absence of Thiamet-G. The cells were treated with MG132 for proteasome inhibition

Then, we validated whether EZH2 is modified by O-GlcNAcylation and the mechanism by which O-GlcNAcylation affects EZH2 function in CRC cell lines. The Co-IP assays showed that exogenous OGT co-precipitated with endogenous EZH2 and that EZH2 was modified by O-GlcNAcylation in the SW480 cells (Fig. 4a–c). Using the YinOYang 1.2 Server (www.cbs.dtu.dk/services/YinOYang), we predicted that EZH2 has nine amino acids with potential for direct O-GlcNAcylation (Supplemental Fig. 2B and C). Because the catalysis of H3K27me3 by EZH2 relies on polycomb repressive complex 2 (PRC2) [35], we further tested whether O-GlcNAcylation affects the stability of the EZH2/PRC2 complex. Co-IP experiments showed that the binding capacity of EZH2 and SUZ12, another indispensable component of PRC2, was increased when the O-GlcNAcylation of EZH2 was upregulated by TMG treatment (Fig. 4d). In addition, O-GlcNAcylation enhanced EZH2 protein stability by prolonging the half-life of degradation (Fig. 4e, f). The close association between O-GlcNAcylation and ubiquitination [18] has been well established, and we further explored whether O-GlcNAcylation enhanced EZH2 protein stability by suppressing its ubiquitination. As shown in Fig. 4g, the ubiquitination level of EZH2 was extremely decreased after the O-GlcNAcylation modification (Fig. 4g), suggesting that O-GlcNAcylation may stabilize EZH2 by suppressing its ubiquitination.

### miR-101 can directly regulate the O-GlcNAcylation level by OGT

**Fig. 5:**
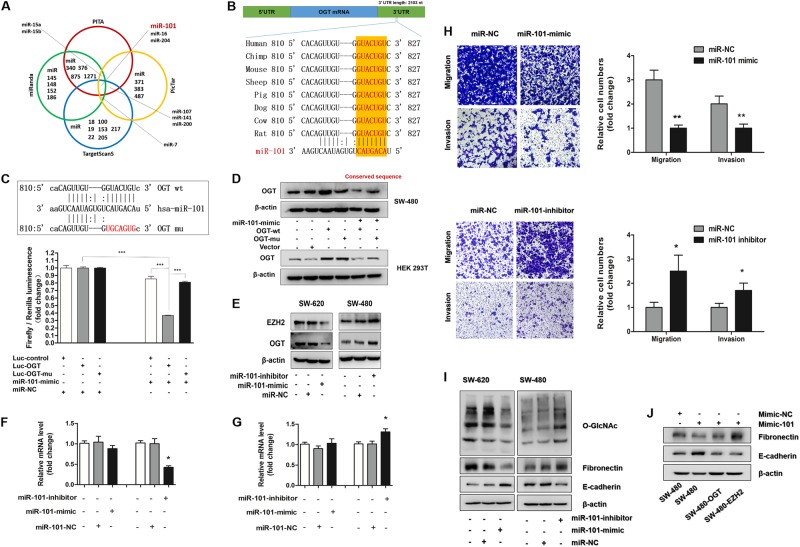
OGT is a direct target of miR-101. **a** Venn diagram displaying miRNAs computationally predicted to target OGT from PITA (red), miRanda (green), TargetScans (blue), and PicTar (orange). **b** Schematic model depicting the binding site of miR-101 in the 3′-UTR of OGT that is highly conserved among several speceies. **c** Schematic of the predicted miR-101-binding sites in the OGT 3′-UTR and mutant binding sites (up). Luciferase activity assay for targeting the 3′-UTR of OGT by miR-101. The wild-type and mutant miR-101 target sequences of OGT were fused to the luciferase reporter and were transfected into the control vector (Luc-OGT and Luc-OGT-mu). Luc-OGT, Luc-OGT-mu, or the control vector was co-transfected with miR-101 or a miRNA-negative control into HEK293T cells, and the luciferase activity was measured. *** represents Student’s t test ***P < 0.001 (down). **d** The expression of OGT was determined by western blotting in SW620 and HEK293T cells. MiR-101 mimic was co-transfected with an OGT wild-type construct (OGT-wt) or an OGT mutant construct with a mutated target sequence of miR-101 (OGT-mu) in SW620 and HEK293T cells. β-Actin was used as the loading control. **e** The expression of EZH2 and OGT was determined by western blotting in SW620 cells transfected with a miR-101 mimic or negative control and SW480 cells transfected with a miR-101 inhibitor or negative control. β-Actin was used as the loading control. **f** mRNA levels of OGT and EZH2 in 13 204 SW620 cells transfected with a miR-101 mimic or negative control. The values shown are 205 expressed as the means ± SEM. * represents Student’s t-test P < 0.05. **g** mRNA levels of OGT and EZH2 in SW480 cells transfected with a miR-101 inhibitor or negative control. The values shown are expressed as the means ± SEM. * represents Student’s t-test P < 0.05. **h** Transwell assay of SW480 or SW620 cells with the indicated treatment. * represents Student’s *t* test *P* < 0.05 and **P < 0.01. **i** Western blotting of O-GlcNAc and EMT markers in SW480 or SW620 cells with the indicated treatment. **j** The expression of EMT markers in SW480 cells was determined by western blotting. MiR-101 mimics were transfected in SW480 cells alone, SW480 cells with OGT overexpression or SW480 cells with EZH2 overexpression. Only the coding sequence (CDS) region of OGT or EZH2, which the target sequence of miR-101 does not recognize, was overexpressed in the SW480 cells. β-Actin was used as the internal control

The above results indicated that O-GlcNAcylation overexpression plays a critical role in promoting CRC metastasis. In addition, TCGA data showed that the increase in the O-GlcNAcylation levels in CRC may be mainly due to an increase in OGT expression. However, the reasons for the OGT increase in CRC are not well understood. MiRNAs have been recently found to play an important role in tumor progression through their function as posttranscriptional regulators. We hypothesized that posttranscriptional regulation by miRNAs may represent an upstream regulatory mechanism of OGT expression. To explore this possibility, we used several web-based target prediction algorithms (TargetScanS, miRanda, pictar, and PITA) to identify miRNAs that could potentially target OGT. MiR-101, miR-16, miR-204, and miR-7 were simultaneously identified by several algorithms as potential regulators of OGT expression (Fig. 5a). In addition, the binding site of miR-101 in the OGT 3′-UTR is highly conserved among several species (Fig. 5b). To further verify that OGT expression is downregulated by miRNAs, a luciferase reporter assay was performed. The luciferase activity of the reporter constructs was most significantly reduced when miR-101 mimic construct, rather than the miR-16, miR-204, or miR-7 mimics, was co-transfected with the OGT 3′-untranslated region (UTR) reporter into HEK293T cells (Fig. 5c, Supplemental Fig. 3A, B and C). Then, a “rescue” experiment further corroborated our hypothesis. We co-transfected miR-101 mimics and a GV146 vector carrying either an OGT expression cassette (OGT-wt) or a mutated seed sequence of miR-101 at the OGT 3′-UTR (OGT-mu) in SW480 or HEK293T cells. The western blotting results revealed that the GV146 vector carrying OGT-mu effectively rescued the miR-101-mediated downregulation of OGT, but the GV146 vector carrying OGT-wt failed (Fig. 5d). Coincidentally, EZH2 is a target of miR-101 (Fig. 5e, Supplemental Fig. 3D and E). As shown in Fig. 5f and g, we also found that miR-101 may regulate the expression of EZH2 by inducing mRNA cleavage and regulates the expression of OGT by suppressing translation. These results strongly suggest that miR-101 is an upstream molecule that regulates OGT expression in CRC.

### miR-101 represses invasion and regulates the EMT in CRC cells by targeting OGT and EZH2

To further verity the function of miR-101 in the migration and invasion of CRC cells, the miR-101 expression levels in human CRC cell lines were examined. As expected, miR-101 was significantly decreased in all cancer cell lines compared with that in the HCoEpiC cell line and was the lowest in the LoVo and SW620 cell lines (Supplemental Fig. 4A). In addition, the expression of miR-101 was negatively linearly correlated with the expression of OGT and O-GlcNAcylation in the CRC cells (Supplemental Fig. 4B). Then, we further explored whether miR-101 regulates the invasion and metastasis of CRC by regulating the expression of OGT and EZH2. As shown in Fig. 5e, the protein levels of OGT and EZH2 were decreased when the SW620 cells were transfected with the miR-101 mimic. Correspondingly, the OGT and EZH2 levels in the SW480 cells were increased following the transfection with the inhibitor (Fig. 5e). In addition, transiently transfecting SW620 cells with miR-101 mimics resulted in the significant inhibition of migration and invasion, and silencing miR-101 in the SW480 cells with an antisense oligonucleotide inhibitor significantly promoted cell migration and invasion (Fig. 5h). These results indicate that miR-101 represses the invasive potential of CRC cells. As O-GlcNAcylation has been shown to promote the EMT in CRC cells, and miR-101 has been predicted to be a potential regulator of O-GlcNAcylation, we hypothesized that miR-101 may exert the opposite effect on the regulation of the EMT in CRC cells. Indeed, western blot analyses showed that the protein level of fibronectin and the O-GlcNAcylation level were decreased following the transfection with the miR-101 mimics in the SW620 cells, whereas E-cadherin was increased (Fig. 5i). Consistent with these results, these markers showed the opposite change following the transfection with the miR-101 inhibitors in the SW480 cells (Fig. 5i). In addition, the overexpression of OGT or EZH2 in the SW480 cells partially blocked the inhibitory effects on the EMT that were induced by the miR-101 mimic transfection (Fig. 5j). These results indicate that miR-101 represses invasion and regulates the EMT in CRC cells by targeting OGT and EZH2.

### O-GlcNAcylation and EZH2 feedback transcriptionally silence miR-101

**Fig. 6:**
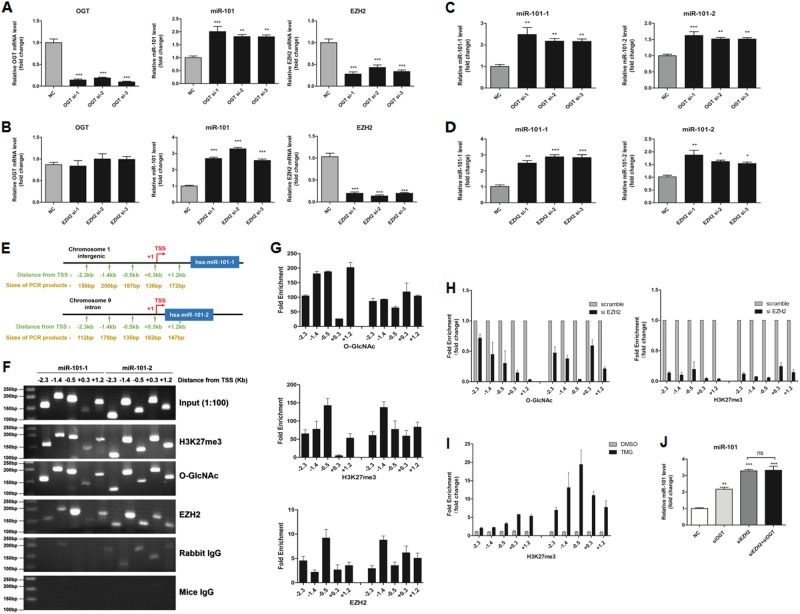
miR-101 is epigenetically silenced by OGT and EZH2 in colorectal cancer cells. **a**, **b** OGT and EZH2 mRNA levels and miR-101 expression levels were assessed by real-time PCR analysis of SW480 cells that had been treated with siScramble (NC), siOGT (**a**), or siEZH2 (**b**) for 72 h. **c**, **d** PremiR-101-1 and premiR-101-2 expression levels were assessed by real-time PCR analysis of SW480 cells that had been treated with siScramble (NC) or siOGT (**c**) or siEZH2 (**d)** for 72 h. The values shown are expressed as the means ± SEM of three independent experiments. **e** Schematic representation of the location of ChIP-qPCR primers in the precursor miR-101-1 and miR-101-2 upstream transcriptional start regions (TSSs) and of the sizes of the PCR products. **f**, **g** ChIP-qPCR assays revealed that H3K27me3, O-GlcNAcylation, and EZH2 bind the TSSs of precursor miR-101-1 and miR-101-2 in SW480 cells. **h** ChIP-qPCR analysis of O-GlcNAcylation and H3K27me3 at miR-101’s promoter regions in SW480 cells that had been treated with siScramble or siEZH2. Normalized O-GlcNAcylation and H3K27me3 levels at the promoter regions of miR-101 in the siEZH2 group are presented relative to those in the siScramble group. The values shown represent the means ± SEM. **i** ChIP-qPCR analysis of H3K27me3 at miR-101’s promoter regions in SW480 cells after 12 h of treatment with TMG (10 μmol/L) or isometric DMSO. Normalized O-GlcNAcylation and H3K27me3 levels at miR-101’s promoter regions in the TMG group are presented relative to those in the DMSO group. The values shown are expressed as the means ± SEM. **j** miR-101 expression levels were assessed by real-time PCR analysis of SW480 cells that had been treated with siScramble (NC), siOGT, siEZH2 or siOGT, and siEZH2 combined for 72 h

Surprisingly, the levels of mature miR-101, precursor miR-101-1 and precursor miR-101-2 were increased when OGT or EZH2 were downregulated (Fig. 6a–d), whereas if EZH2 was silenced in advance, no significant changes were observed when OGT was downregulated (Fig. 6j), indicating that OGT and EZH2 may feedback to transcriptionally silence miR-101. Similarly, the levels of mature miR-101, precursor miR-101-1 and precursor miR-101-2 were correspondingly decreased when O-GlcNAcylation was upregulated in the SW480 cells (Supplemental Fig. 5A, B and C). On the basis of our previous finding that EZH2 feedback regulates miR-101 in hepatocellular carcinoma [38], we hypothesized that O-GlcNAcylation provides feedback that regulates miR-101 by H3K27me3 in CRC. To test our hypothesis, we conducted ChIP-qPCR analysis of the transcriptional start regions (TSSs) of miR-101-1 and miR-101-2 precursors. The results showed that the miR-101 promoter regions are highly enriched in EZH2, H3K27me3, and O-GlcNAcylation, and the binding peaks are near the TSSs in the SW480 (Fig. 6e–g) and SW620 (Supplemental Fig. 6A, B and C) cells. Meanwhile, the EZH2 knockdown in the SW480 cells almost completely eliminated the enrichment of O-GlcNAcylation and H3K27me3 in these regions (Fig. 6h), and when O-GlcNAcylation was upregulated, the H3K27me3 enrichment in these regions was significantly increased (Fig. 6i). Meanwhile, the knockdown of EZH2 or OGT in the SW620 could eliminated the enrichment of O-GlcNAcylation or H3K27me3 in the miR-101 TSS regions, respectively (Supplemental Fig. 6D and E). These results support the hypothesis that O-GlcNAcylation feedback negatively regulates miR-101 and that EZH2 is a necessary component of this type of regulation.

## Discussion

It has been well documented that the excessive caloric intake and hyperglycemia associated with a modern lifestyle constitute important CRC risk factors and significantly promote CRC progression, including metastasis [2, 39]. An increase in O-GlcNAcylation may be a possible cause of this phenomenon. Excessive nutrient intake and hyperglycemia are believed to feed the HBP and promote abnormally increased levels of O-GlcNAcylation in colorectal epithelial cells [30]. In addition, owing to the Warburg effect, cancer cells require higher glucose uptake for energy and metabolic intermediate production to support cell survival and metastasis [30]. As a consequence, O-GlcNAcylation levels are generally increased in CRC and have been recognized as a lynchpin for the development and progression of CRC [11–13]. Studies have shown that CRC patients with hyperglycemia or diabetes are at a higher risk for metastasis [40–43].

In breast cancer and fatty liver-associated liver cancer, O-GlcNAcylation was previously thought to be a central component linking metabolism to invasion and metastasis via the SIRT1/FOXM1 axis [44, 45]. O-GlcNAcylation also enhances ovarian cancer cell migration by decreasing the expression of EMT-related proteins [46]. Although certain studies have identified that O-GlcNAcylation and/or OGT are highly expressed in metastatic CRC and other types of cancer tissue [47–49], the molecular mechanisms of how O-GlcNAcylation promotes CRC metastasis were unclear. In addition, notably, this finding is seemingly inconsistent with a recent study conducted by Parunya Chaiyawat and Galit Yehezkel in which OGT was not higher in SW620 than SW480. This discrepancy may be explained by the fact that two different culture mediums were used. The level of OGT expression in cancer cells has been shown to be very sensitive to changes in the nutrient status of the medium, especially changes in glucose [11]. To exclude the influence of the culture conditions, all cells in our research were cultured in Dulbecco's Modified Eagle's medium (DMEM) basic (supplemented with 25 mM d-glucose, 1 mM sodium pyruvate, and 4 mM l-glutamine) with 10% FBS and 1% penicillin/streptomycin, and the protein and mRNA levels of OGT were found to be slightly increased in SW620, which is inconsistent with those two papers. Meanwhile, the protein and mRNA levels of OGA were decreased in SW620. Intriguingly, negative feedback mechanisms appear to exist between OGT and OGA to buffer large changes in the levels of O-GlcNAcylation [7]. Slawson et al. showed that in addition to elevating the O-GlcNAcylation level, the pharmacological inhibition of OGA could decrease the OGT level and elevate the OGA level. In contrast, pharmacologically lowering the O-GlcNAc levels also results in higher OGT expression and lower OGA expression [50]. These changes in the patterns of OGT and OGA expression are consistent with feedback signals that attempt to ease the rapid and fierce change in the O-GlcNAc levels, although the mechanisms underlying such a feedback mechanism are unknown. Despite these negative feedback mechanisms that attempt to “normalize” the changing O-GlcNAc levels, in our study, the colon cancer cells maintained a strikingly elevated level of O-GlcNAcylation, and the cell lines with high metastatic potential exhibited higher expression levels of O-GlcNAcylation than the other CRC cell lines.

Following the inhibition of OGA in SW480, Nearly, Isam Khalaila et al. [51] observed a global elevation of protein O-GlcNAcylation and an increase in the expression of E-cadherin. Although the authors also observed an increase in O-GlcNAcylation to promote the EMT and metastasis in CRC, the expression of E-cadherin appears to contradict our conclusion. In our research, we found that the total E-cadherin level in SW480 was increased after the TMG treatment (data not shown), whereas membranous E-cadherin was decreased. Multiple posttranslational modifications are known to regulate the location and function of E-cadherin [52]. Meanwhile, O-GlcNAcylation prevents E-cadherin trafficking to the membrane and blocks its binds to p120 catenin [53]. We surmise that the following two points are the main reasons for this phenomenon. First, the O-GlcNAc modification of E-cadherin could block its cell surface transport. Therefore, the elevated total E-cadherin level is likely mainly located in the cytoplasm and not the membrane. Second, the O-GlcNAcylation of E-cadherin could block its ability to bind p120 catenin, which results in reduced intercellular adhesion and, thus, stimulates migration. Furthermore, our study not only reaffirmed the increased expression of O-GlcNAcylation in metastatic CRC but also further partially revealed the reasons why O-GlcNAcylation and OGT are increased in CRC. In addition, we explored the role of O-GlcNAcylation in conferring CRC cell metastasis through the miR-101/O-GlcNAcylation/EZH2 signaling regulatory feedback circuit.

Epigenetic regulation is associated with specific histone modifications. EZH2, a histone methyltransferase, is a necessary enzyme for the H3K27me3 activity of PRC2 at target gene promoters, leading to epigenetic silencing. Increased EZH2 expression or activity is a marker of advanced and metastatic disease in many solid tumors [35, 36, 54]. Importantly, drugs that inhibit EZH2 activity, such as tazemetostat, are in clinical trials, and these trials have shown favorable preliminary effects [35, 55]. Tiwari N et al. [36] discovered that EZH2-mediated H3K27me3 is associated with key EMT genes, representing an epigenetic EMT signature in breast cancer cells. In this study, we found that the ubiquitination level of EZH2 was extremely decreased and that the binding capacity of EZH2 and SUZ12 was upregulated when the O-GlcNAcylation of EZH2 was increased by TMG treatment. However, the precise mechanism by which O-GlcNAcylation inhibits the ubiquitination of EZH2 and regulates the stability of PRC2 requires further investigation. O-GlcNAcylation is known to modulate protein stability and protein–protein degradation interactions. For example, on p53, O-GlcNAcylation at Ser 149 is associated with decreased Thr155 phosphorylation, which results in decreased p53 ubiquitination [56]. In addition, the modification of Snail1 at Ser112 prevents degradation by decreasing the ubiquitination of this protein [57]. EZH2 may have a similar regulatory mechanism. In fact, EZH2 can be phosphorylated at Thr345 or Thr487 by cyclin-dependent kinase 1 [58], thus leading to its ubiquitination and subsequent degradation by the proteasome [58, 59]. In addition, Chu CS et al. [60] verified that EZH2 is O-GlcNAcylationylated at Ser75, and we also predicted nine O-GlcNAcylation sites in EZH2 that may be directly modified by O-GlcNAcylation (Supplemental Fig. 2B and C).

In addition to metabolism, we found that miRNA dysregulation may partially explain the increase in O-GlcNAcylation in CRC. TCGA data suggest that the increase in the O-GlcNAcylation levels in CRC may be mainly due to an increase in OGT expression. In addition, MiR-101 was identified as a potential regulator of OGT, and its level was significantly decreased in five cancer cell lines and CRC tissues. Interestingly, EZH2 is also one of the targets of miR-101, indicating that the decrease in miR-101 in CRC may promote metastasis by directly regulating both OGT and EZH2. MiRNAs constitute a class of small non‑coding RNA molecules that play regulatory roles at the posttranscriptional level by suppressing the translation of protein‑coding genes or inducing mRNA cleavage [61]. As shown in Fig. 5f and g, we found that miR-101 may regulate the expression of EZH2 by inducing mRNA cleavage and regulates the expression of OGT by suppressing translation. Surprisingly, we also found that O-GlcNAcylation/EZH2 feedback transcriptionally silenced miR-101. The levels of mature miR-101, precursor miR-101-1, and precursor miR-101-2 were correspondingly increased when OGT or EZH2 were downregulated (Fig. 6h, i), whereas if EZH2 was silenced in advance, no significant change was observed when OGT was downregulated (Fig. 6j), indicating that OGT feedback may transcriptionally silence miR-101 in an EZH2-dependent manner. Meanwhile, miR-101 feedback may transcriptionally silence itself as a single transient transfection of mature miR-101 mimic efficiently inhibited the expression of premiR-101-1 and premiR-101-2 (Supplemental Fig. 5D). H3K27me3 modification at target gene promoters is known to lead to epigenetic silencing. Hyper-O-GlcNAcylation promotes EZH2 protein function, and EZH2 is a key enzyme that contributes to H3K27me3. For these reasons, we hypothesized that O-GlcNAcylation feedback may regulate miR-101 by H3K27me3. Indeed, the ChIP-qPCR assays verified that the miR-101 promoter regions are highly enriched in EZH2, H3K27me3, and O-GlcNAcylation. Recently, Guo et al. [62] also found that O-GlcNAcylation and H3K27me3 were simultaneously enriched in the promoter regions of multiple genes.

In CRC cells, the protein expression of OGT and EZH2 was inversely correlated with the miR-101 level, and EZH2 expression was positively correlated with the O-GlcNAcylation level. However, in CRC patients, the existence of these expression patterns is uncertain. We analyzed EZH2 and OGT protein expression levels by western blotting and the miR-101 level by real-time PCR in 30 freshly collected CRC tissues and adjacent normal tissues. The results verified that OGT and EZH2 protein expression had a negative relationship with the miR-101 level in the tissues of CRC patients (Supplemental Fig. 7A, B and C). Then, the levels of O-GlcNAcylation, OGT, and EZH2 were investigated using IHC in 100 CRC tissues (Supplemental Fig. 7D) that were divided into three groups (weak, medium, or strong) according to the IHC score (Supplemental Fig.7E and F). According to the Pearson correlation analysis, the results also showed that there was a positive correlation among the levels of OGT, EZH2, and O-GlcNAcylation in vivo (Supplemental Fig. 7G). Our in vitro results support the hypothesis that dysregulation of the miR-101/O-GlcNAcylation/EZH2 signaling regulatory feedback circuit promotes CRC metastasis; however, further investigations are required to fully verify this feedback circuit at the clinical level.

**Fig. 7:**
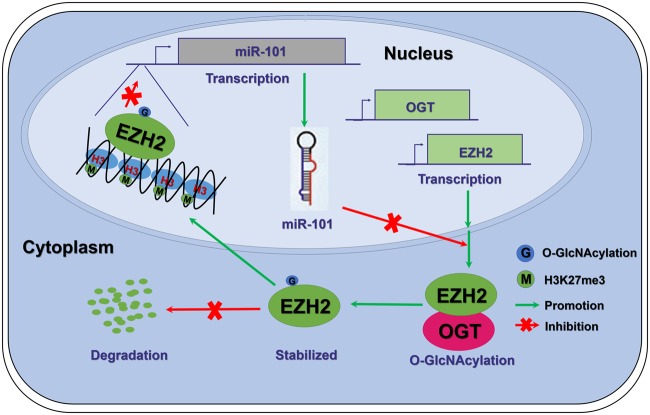
Schematic model depicting that miR-101/O-GlcNAcylation/EZH2 signaling promotes metastasis in CRC. In colorectal cancer tissues, a low miR-101 level leads to an increase in the protein levels of OGT and EZH2. Meanwhile, OGT enhances the stability and function of EZH2 by O-GlcNAcylation. As a result, EZH2 promotes the tri-methylation of H3K27 in the miR-101 promoter region, which further reduces the level of miR-101 in cancer tissues. In brief, the miR-101-O-GlcNAc/EZH2 regulatory feedback circuit promotes metastasis in CRC

In summary, in CRC cells, miR-101/O-GlcNAcylation/EZH2 signaling forms a feedback loop that promotes metastasis. The downregulation of miR-101 in CRC promotes the elevation of O-GlcNAcylation and, thus, enhances EZH2 protein stability and function, which, in turn, further reduces the expression of miR-101 (Fig. 7). This findings provide a new mechanistic insight into the basic theory of cancer metastasis and suggest that blocking the O-GlcNAcylation may represent a potential therapeutic strategy for metastatic CRC.

## Materials and methods

### Clinical samples

Three CRC tissue microarrays (HCol-Ade180Sur-09) were purchased from Shanghai Outdo Biotech, which contain 80 cases of gastric adenocarcinoma with paired paraneoplastic tissues, with one point for each tissue, and 20 cases of unpaired cancer tissues. In addition, 30 cases of primary CRC tissues with paired adjacent normal tissues samples were obtained from patients who had undergone CRC surgery at Xijing Hospital of Digestive Diseases. All samples were clinically and pathologically verified. This study was approved by Xijing Hospital’s Protection of Human Subjects Committee. Informed consent was obtained from each patient.

### Cell culture

The human CRC cell lines LoVo, SW620, SW480, HCT-116, and HT-29 were purchased from ATCC. The normal colon epithelial cell line HCoEpiC was purchased from ScienCell. All cells were cultured in DMEM basic (supplemented with 25 mM d-glucose, 1 mM sodium pyruvate, and 4 mM l-glutamine, Gibco) with 10% fetal bovine serum (Bioind) and 1% penicillin–streptomycin (Gibco). All cells were cultured at 37 °C with 5% (vol/vol) CO_2_. All cells were tested for mycoplasma contamination.

### Lentivirus

Virus packaging was performed in HEK293T cells by co-transfection with lentiviral vectors with the packaging plasmid pHelper 1.0 vector (GeneChem Co., Ltd., Shanghai, China) and the envelope plasmid pHelper 2.0 vector (GeneChem Co., Ltd.) using Lipofectamine 2000 (Invitrogen). At 48 h after transfection, supernatants containing lentiviral particles were collected, and the virus titer was quantified according to the manufacturer’s instructions. Lentiviral vectors encoding short hairpin RNAs (shRNAs) (Sh-1: ATTATTGTAACCACCCGTT; Sh-2 TGAGCAGTATTCCGAGAAA; Sh-3: CAATCATTTCATTGATCTT) targeting OGT were generated using the GV344 vector (hU6-MCS-Ubiquitin-Luc_firefly IRES-puromycin, GeneChem Co., Ltd., Shanghai, China). A scrambled GV344 vector (TTCTCCGAACGTGTCACGT) was used as the negative control. Stable transfectants overexpressing OGT were generated by lentiviral transduction using a GV341 vector (Ubi-MCS-3FLAG-SV40-puromycin, GeneChem Co., Ltd.). An empty vector was used as the negative control. Stable transfectants overexpressing EZH2 were generated by lentiviral transduction using a GV367 vector (Ubi-MCS-SV40-EGFP-IRES-puromycin, GeneChem Co., Ltd.). An empty vector was used as the negative control.

### Plasmids

Human full-length OGT (NM_181672) with wild-type or mutated seed sequences of miR-101 at the OGT 3′-UTR were constructed by cloning the open reading frames and downstream 3’-UTRs into the pcDNA 3.1 vector GV146 (CMV-MCS-IRES-EGFP-SV40-Neomycin, GeneChem Co., Ltd., Shanghai, China). The 3′-UTR fragments of OGT and EZH2 containing the miR-101 putative target sites were amplified and were cloned between XhoI and NotI sites downstream of the SV40 promoter-driven Renilla luciferase cassette in the psiCHECK-2 vector (Promega). To mutate the miR-101-binding sites of these vectors, a site-directed mutagenesis kit (Agilent Technologies) was used according to the manufacturer’s instructions. The miR-101-2-5p sequences were amplified and were cloned into the pGC-FU vector (GeneChem Co., Ltd., Shanghai, China) between XhoI and BamHI sites for expression driven by the CMV promoter.

### Dual-luciferase reporter assay

When cells reached 60% confluence in 24-well plates, they were transfected using X-tremegene HP (Roche). A firefly luciferase reporter gene construct (0.1 μg), miRNA construct (0.4 μg), and Renilla luciferase construct (0.02 μg) were co-transfected in well. In addition, 48 h after transfection, luciferase activity was measured using the Dual-Luciferase Reporter Assay System (Promega) according to the manufacturer’s instructions.

### Statistical analysis

We used Prism 5 (San Diego, CA, USA) and SPSS 18.0 (Chicago, IL, USA) software for statistical analyses. All values are expressed as the means ± SD unless otherwise indicated. The differences between the two groups were assessed using Student’s *t* test. A *P* value < 0.05 was considered to indicate a significant difference. Before performing Student’s *t* test, we performedcdk a variance homogeneity test and normality test for the variable data. Overall survival curves were estimated by the Kaplan–Meier method and Cox proportional hazards model. Two-tailed Friedman rank-sum tests were performed to analyze the O-GlcNAcylation level in 15 cases of adjacent normal tissues, tumor tissues, and positive metastatic lymph nodes, and *P* < 0.05 was considered statistically significant. No statistical method was used to predetermine the sample size.

## Electronic supplementary material

Supplemental files

Supplemental figure 1

Supplemental figure 2

Supplemental figure 3

Supplemental figure 4

Supplemental figure 5

Supplemental figure 6

Supplemental figure 7

List of LC-MSMS
