# Supplementary material for: O-GlcNAcylation promotes colorectal cancer metastasis via the miR-101-O-GlcNAc/EZH2 regulatory feedback circuit
Source: Oncogene. 2018 Aug 9;38(3):301–16. doi: 10.1038/s41388-018-0435-5 (PMC6336687; doi:10.1038/s41388-018-0435-5)
Supplement: Supplementary file 1 — Supplemental files [file 41388_2018_435_MOESM1_ESM.pdf]

Supplemental Experimental Procedures

Western blot analysis

The cells were washed with PBS three times and collected in RIPA lysis buffer (Beyotime Biotechnology, Shanghai, China) supplemented with protease inhibitor cocktail (Calbiochem, San Diego, USA) and phosphatase inhibitor cocktail (Calbiochem, San Diego, USA). The protein concentration was determined by Coomassie brilliant blue (Beyotime Biotechnology, Shanghai, China) staining. The membranous protein extraction of Claudin 7, E-cadherin and Na-K-ATPase was performed using a Qproteome® Plasma Membrane Protein Kit (QIAGEN, Cat: 37601, Hilden, Germany). After electrophoresis, the proteins were transferred to a polyvinylidene difluoride membrane (Merck Millipore, Darmstadt, Germany). After blocking with Tris-buffered saline with 0.1% Tween 20 (TBS-T) containing 5% skim milk for 1 hour at RT, primary monoclonal antibody was added to the membrane and was incubated overnight at 4°C. The next day, the membranes were incubated with corresponding secondary antibodies for 1 hour at RT, and the signals were detected in a Bio-Rad ChemiDoc XRS imaging system. The ratio of the gray value of the target protein to that of β-actin represented the relative amount of protein.

Table 1: The list of primary antibodies used

Target	Usage	Source	Catalog number	Dilution
O-GlcNAc	WB	Abcam	Ab2739	1:1000
EZH2	WB	CST	5246	1:1000
OGT	WB	CST	5368	1:1000
E-cadherin	WB	CST	3195	1:1000

Claudin 7	WB	Abcam	Ab27487	1:1000
Na-K-ATPase	WB	Abcam	Ab185210	1:1000
Vimentin	WB	Millipore	MABT121	1:1000
Fibronectin	WB	Abcam	2413	1:1000
H3	WB	Abcam	1791	1:1000
H3K27me3	WB	CST	9733	1:1000
Snail-1	WB	Abcam	ab53519	1:1000
Flag	WB	Sigma-Aldrich	SAB4200071	1:2000
Ubiquitin	WB	Abcam	Ab134953	1:1000
β-actin	WB	Sigma-Aldrich	A2228	1:5000

Quantitative real-time polymerase chain reaction (qRT-PCR)

Total RNA was extracted from samples using the TaKaRa MiniBEST Universal RNA Extraction kit (TaKaRa, Tokyo, Japan). The RNA extract was measured using an ultraviolet spectrophotometer at 260 and 280 nm. Only samples with an OD260/OD280 ratio of 1.8 to 2.0 were used for subsequent analyses. Complementary DNA was obtained by reverse transcription according to the manufacturer's instructions (TaKaRa, Tokyo, Japan). The mRNA level expression was analyzed by a real-time fluorescence quantitative PCR instrument with CFX96 software (Bio-Rad, CA, USA) according to the manufacturer's instructions. The cycling parameters were as follows: 95°C for 30 seconds, followed by 45 cycles of 95°C for 5 seconds and 60°C for 30 seconds. A melting curve analysis was then performed. The relative mRNA level was normalized to that of β-actin.

Table 2: The list and sequences of primers used for qRT-PCR

Primer	Sequence (5'-3')
OGT-Forward	AGAAGGGCAGTGTTGCTGAAG
OGT-Reverse	TGATATTGGCTAGGTTATTCAGAGAGTCT
OGA-Forward	GCGGTGTGGTGGAAGGATT
OGA-Reverse	CCATTTCTGGAGCCTTCTAAAGAG
EZH2-Forward	TAATGTGCTGGAATCAAAGGATAC
EZH2-Reverse	GCTTCATCTTTATTGGTGTTTGAC
E-cadherin-Forward	GAGTGCCAACTGGACCATTCAGTA
E-cadherin-Reverse	AGTCACCCACCTCTAAGGCCATC
Fibronectin-Forward	GCCAGATGATGAGCTGCAC
Fibronectin- Reverse	GAGCAAATGGCACCGAGATA
Vimentin-Forward	CAGGCAAAGCAGGAGTCCAC
Vimentin -Reverse	GCAGCTTCAACGGCAAAGTTC

Cell migration and invasion assay

Cell migration and invasion was assayed using Transwell chambers (6.5 mm; Corning, NY, USA) with 8-μm pore membranes; for cell invasion assays, the upper face of the membrane was covered with 70 μl of Matrigel (1 mg/ml) (BD Biosciences, NJ, USA). The lower chamber was filled with 1500 μl of lower medium (medium with 20% FBS). The cells (5×10^4 cells/well) were suspended in 200 μl of upper medium (medium with 1% FBS) and were plated into the upper chamber. After 12 and 24 hours, the number of crystal violet-stained cells on the undersurface of the polycarbonate membranes was visually counted in five random fields at 100× magnification.

Three-dimensional spheroid BME cell invasion assay

Three-dimensional (3D) spheroid basal membrane extract (BME) cell invasion

assays were performed using the Cultrex® 96-well 3D Spheroid BME Cell Invasion assay kit (Sigma-Aldrich, 3500-096-K) according to the manufacturer's instructions. Briefly, 3000 cells were resuspended in 50 μl 1x Spheroid Formation ECM and added to a 96 Well Spheroid Formation Plate. Then, the plate was centrifuged at 200 x g for 3 minutes at room temperature and incubated at 37°C in an incubator for 72 hours to promote spheroid. While on ice, 50 μl of Invasion Matrix were added per well, followed by centrifugation at 300 x g at 4°C for 5 minutes. The plate was transferred to a 37°C incubator for 1 hour to promote gel formation on the Invasion Matrix, and 100 μl warm cell culture medium was added per well after 1 hour. The plate was incubated at 37°C in an incubator for 7days, and the spheroid in each well was photographed every 24 hours using a 4x objective. The images were analyzed using Image J software, and the changes in the area of the invasive structure were measured to determine the extent of the 3D culture BME cell invasion in each sample.

Transfection of short interfering RNA (siRNA) or miRNA mimics/inhibitors

Double-stranded siRNA targeting human OGT (purchased from Biomics Biotechnologies Co., Ltd., Nantong, China), Opti-MEM (Invitrogen, Karlsruhe, Germany) media and HiPerFect® transfection reagent (Qiagen, Dusseldorf, Germany) were mixed together and were incubated according to the manufacturer's instructions. In all experiments, scrambled siRNA served as a control. The cells were analyzed at 48 hours posttransfection.

Table 3: The list and sequences of primers used for transfection

**Primer**	**Sequence (5'-3')**

OGT-si 1 Forward	CGCGUGCCAUCCAAAUUAAdTdT
OGT-si 1 Reverse	UUAAUUUGGAUGGCACGCGdTdT
OGT-si 2 Forward	GCACGGCUCUGAAACUUAAdTdT
OGT-si 2 Reverse	UUAAGUUUCAGAGCCGUGCdTdT
OGT-si 3 Forward	GGCAGAAGCUUAUUCGAAUdTdT
OGT-si 3 Reverse	AUUCGAAUAAUAAGCUUCUGCCdTdT
EZH2-si 1 Forward	CAGCUCUAGACAACAAACCdTdT
EZH2-si 1 Reverse	GGUUUGUUGUCUAGAGCUGdTdT
EZH2-si 2 Forward	ACAGAAGAGGGAAAGUGUAdTdT
EZH2-si 2 Reverse	UACACUUUCCCUCUUCUGUdTdT
EZH2-si 3 Forward	UGCCCUUGGUCAAUAUAAUdTdT
EZH2-si 3 Reverse	AUUAUAUUGACCAAGGGCAdTdT
miR-101 mimic	Syn-hsa-miR-101 miScript miRNA Mimic (QIAGEN MSY0000099)
miR-NC mimic	AllStars Neg. Control siRNA (QIAGEN 192058331)
miR-101 inhibitor	Anti-hsa-miR-101 miScript miRNA Inhibitor (QIAGEN MIN0000099)
miR-NC inhibitor	miScript Inhibitor Neg. Control (QIAGEN 188304225)

Immunohistochemical (IHC) staining

Monoclonal antibodies against OGT (CST, No. 5368), O-GlcNAc (Abcam, Ab2739) and EZH2 (CST, No. 5246) were assessed. Tissue staining was performed as previously published[1]. On each slide, both the IHC staining score of positive cells and the intensity of the positive cells were calculated using the semiquantitative scoring method. Evaluation of immunostaining intensity was performed as previously described[2].

Chromatin immunoprecipitation (ChIP)

Chromatin immunoprecipitation was performed using the EZ-Magna ChIP™ A/G (Millipore, 17-10086) according to the manufacturer's instructions. Real-time PCR was conducted using different sets of primers to amplify the miR-101 promoter

regions in the immunoprecipitated DNA, as well as in the input DNA. The cycling parameters were as follows: 94°C for 10 minutes, followed by 50 cycles of 94°C for 20 seconds and 60°C for 1 minute. A melting curve analysis was then performed. The amplified fragments were then analyzed using agarose gel electrophoresis. Prior to immunoprecipitation, 1% of the chromatin was reserved for use as the input control, and a nonspecific antibody against IgG (Millipore) served as the negative control. The primer sequences and antibodies for the analyzed promoters are provided below.

Table 4: The list and sequences of primers used for ChIP

Primer	Sequence (5’-3’)
**miR-101-1:**	
-2.3kb Forward	TGACAATCCAGATCCTCCTCTGAGC
-2.3kb Reverse	GACTGGCGGTTGAGCAGGCA
-1.4kb Forward	GGCATGGCCCTGGCCTCAAAG
-1.4kb Reverse	GGGGTGGGGACGGGACTCAC
-0.5kb Forward	GGCGGACAGGCGAGTGAAGG
-0.5kb Reverse	GAAAGGAGGCTCCGGCAGCG
+0.3kb Forward	TTCCCGGTCATGAGACCCGG
+0.3kb Reverse	CCACAACGTGACCGTCGCCA
+1.2kb Forward	TGGGGTGTCAGTGGCAACGC
+1.2kb Reverse	TTTACAGTCGCCGCCGGACG
**miR-101-2:**	
-2.3kb Forward	TCCAGCCCCTCTACTCGGCATT
-2.3kb Reverse	TGAGGCCAGATCGTGACTGC
-1.4kb Forward	CCACTGGCTCTCTGCATTTCTAGC
-1.4kb Reverse	CCCAATTAAGGCAAGCCTTTCCG
-0.5kb Forward	AGGCTAGCAGTAAGATCAAT
-0.5kb Reverse	TCCTTTTTAAGGCATGTAGG

+0.3kb Forward	AGGAAAAGGGGGTGGAAGGCAT
+0.3kb Reverse	CGGCCTTTTCATGGCCTTAC
+1.2kb Forward	GTTTCCAGGTTAGAGGTAAACTCC
+1.2kb Reverse	TCCCAGTGAGCTCATATCCT

Table 5: The list of primary antibodies used for ChIP

Target	Usage	Source	Catalog number	Dilution
EZH2	ChIP	CST	5246	4 ug
O-GlcNAc	ChIP	Abcam	Ab2739	4 ug
H3K27me3	ChIP	CST	9733	4 ug
IgG (mouse)	ChIP	Millipore	NI03-100UG	4 ug
IgG (rabbit)	ChIP	Millipore	NI01-100UG	4 ug

Immunofluorescence assay

Cells were plated onto glass coverslips, fixed with 4% paraformaldehyde for 15 minutes and permeabilized with 0.1% Triton X-100 in PBS for 10 minutes. Blocking solution was applied for 1 hour at room temperature. Primary antibodies were applied at 4°C overnight. Alex Fluor 488-conjugated or Alexa Fluor 594-conjugated secondary antibodies were loaded and were incubated for 2 hours at room temperature. Immunostaining signals and DAPI-stained nuclei were visualized at room temperature using a confocal microscope and Fluoview software.

Table 6: The list of primary antibodies used for Immunofluorescence assay (IF)

Target	Usage	Source	Catalog number	Dilution
EZH2	IF	CST	5246	1:200
O-GlcNAc	IF	Abcam	Ab2739	1:200
OGT	IF	Abcam	ab198530	1:200
E-Cadherin	IF	CST	3195	1:200

Vimentin	IF	Millipore	MABT121	1:200

miRNA expression analysis and miScript precursor assay

Total RNA was extracted from samples using a miRNeasy Mini kit (Qiagen, No. 217004) according to the manufacturer's instructions. For miRNA expression analysis, reverse transcription was performed using Mir-X™ miRNA First-Strand Synthesis kit (TaKaRa, No. 638313), and the cycling parameters of quantitative, real-time PCR were as follows: 95°C for 30 seconds, followed by 45 cycles of 95°C for 5 seconds and 60°C for 30 seconds. A melting curve analysis was then performed. The relative amount of miRNA was normalized to that of U6. For the miScript precursor assay, reverse transcription was performed using a miScript II RT kit (Qiagen, No. 218161) and quantitative, real-time PCR was performed using a miScript SYBR Green PCR kit (Qiagen, No. 218073) with the cycling parameters as follows: 95°C for 15 minutes, followed by 45 cycles of 94°C for 15 seconds, 55°C for 30 seconds and 70°C for 30 seconds. A melting curve analysis was then performed. The relative amount of miRNA was normalized to U6.

Table 7: The list and sequences of primers used for miRNA or precursor expression analysis

Primer	Sequence (5'-3')
MiR-101	TGCGGTACTGTGATAACTGAA
Universal Primer	Takara 1609256A
U6-F	Takara 1609257A
U6-R	Takara 1609258A
pre-miR-101-1	QIAGEN MP00000091
pre-miR-101-2	QIAGEN MP00000098

Co-immunoprecipitation (co-IP) and mass spectrometry (MS)

Co-immunoprecipitation was performed using the Pierce® Co-Immunoprecipitation kit (Thermo scientific 26149) according to the manufacturer's instructions. Fractions of the bed volume were collected and were resolved on SDS-polyacrylamide gels, stained with Coomassie brilliant blue, and subjected to LC-MS/MS sequencing and data analysis.

Table 8: The list of primary antibodies used for Immunoprecipitation

Target	Usage	Source	Catalog number	Dilution
EZH2	IP	CST	5246	2 ug
O-GlcNAc	IP	Abcam	Ab2739	2 ug
Flag	IP	Sigma-Aldrich	SAB4200071	2 ug
IgG (mouse)	IP	Millipore	NI03-100UG	2 ug
IgG (rabbit)	IP	Millipore	NI01-100UG	2 ug

Mice:

In total, 5×10^6 SW620 cells expressing shRNAOGT2, shRNAOGT3 or shRNA-Ctrl vector or SW480 cells expressing OGT-vector or empty vector were injected into nude mice (5-week-old male nude mice with a body weight of 18–22 g) via the lateral tail vein, and ten nude mice were injected for each experiment. All the BALB/c nude mice were maintained under specific pathogen-free conditions. This study was carried out in strict accordance with recommendations in the Guide for the Care and Use of Laboratory Animals of the National Institutes of Health. All experimental procedures were approved by the Institutional Review Board of Xijing Hospital. The animal experiments were carried out with the approval of the Institutional Committee for Animal Research and in agreement with national guidelines for the care and use of laboratory animals.

References:

1 Jiang M, Qiu Z, Zhang S, Fan X, Cai X, Xu B *et al*. Elevated O-GlcNAcylation promotes gastric cancer cells proliferation by modulating cell cycle related proteins and ERK 1/2 signaling. Oncotarget 2016.

2 Zhou L, Shang Y, Liu C, Li J, Hu H, Liang C *et al*. Overexpression of PrPc, combined with MGr1-Ag/37LRP, is predictive of poor prognosis in gastric cancer. International journal of cancer Journal international du cancer 2014; 135: 2329-2337.

Supplemental Figure Legends:

Supplemental Figure 1: 3D Spheroid BME cell invasion assay of SW620 cells (Vector, Sh-2, or Sh-3) **(A)**, SW480 cells (treated with PUGNAc, TMG or isometric DMSO) **(B)** and SW480 cells (treated with isometric DMSO, Thiamet-G (10 μmol/L) or Thiamet-G (10 μmol/L) and GSK-343 (3 nmol/L) combined) **(C)**. 3D Spheroid BME cell invasion assay of photographs (4X objective) of all spheroids in each well for 7 days. Quantitative analysis of the surface area for all spheroids. Normalized areas for all spheroids are presented relative to the area on the first day. All the results are presented as the means ± SEM of three independent biological replicates. * represents Student's t-test *P < 0.05, **P < 0.01, ***P < 0.001.

Supplemental Figure 2: (A) Confocal analysis of the O-GlcNAcylation (green), OGT (green) and EZH2 (Red) level in SW-480 cells treated with or without TMG. Nuclei were stained with 4′,6-diamidino-2-phenylindole (DAPI; blue). **(B, C)** The O-GlcNAc sites positively predicted by the YinOYang 1.2 server (www.cbs.dtu.dk/services/YinOYang) are shown with red residues at the top. Ser/Thr residues that are predicted to be O-GlcNAcylated and phosphorylated are marked by a blue asterisk (*) in both the residue and the YinOYang column. The green vertical lines show the O-GlcNAc potential of Ser/Thr residues, and the red horizontal wavy line indicates the threshold for modification potential. "+" means O-GlcNAc potential > Thresh-1; "++" means O-GlcNAc potential > Thresh-2 (Thresh-2 is a threshold based on more stringent surface measures); "+++" means O-GlcNAc potential > (Thresh-2 + 0.1). **(D)** Western blotting for H3K27me3 and H3 in SW-480 cells after 3 days of treatment with GSK343 (3 μmol/L) or isometric DMSO.

Supplemental Figure 3: (A, B, C) Schematic of the predicted miR-7 (a), miR-16 (b) or miR-204 (c) binding sites in the OGT 3'UTR and binding sites mutant (up). Luciferase activity assay for targeting the 3′-UTR of OGT by miR-7 (A), miR-16 (B) or miR-204 (C). The wild-type and mutant miR-7 (A), miR-16 (B) or miR-204 (C) target sequences of OGT

were fused to the luciferase reporter and were transfected into the control vector (Luc-OGT and Luc- OGT -mu). Luc-OGT, Luc-OGT-mu or the control vector was co-transfected with miR-7 (A), miR-16 (B), miR-204 (C) or a miRNA negative control into HEK293T cells, and the luciferase activity was measured. * represents Student’s t-test *P < 0.05, **P < 0.01 and ***P < 0.001. (down). **(D)** Schematic of two predicted miR-101 binding sites in the EZH2 3’UTR and in the binding site mutant. **(E)** Luciferase activity assay for targeting the 3′-UTR of EZH2 by miR-101. The wild-type and mutant miR-101 target sequences of EZH2 were fused to the luciferase reporter and were transfected into the control vector. Luc-EZH2-wt, Luc-EZH2-mu 1, Luc-EZH2-mu 2, Luc-EZH2-mu 1+2 or the control vector was co-transfected with miR-101 or a miRNA negative control into HEK293T cells, and the luciferase activity measured. *** represents Student’s t-test ***P < 0.001.

Supplemental Figure 4: (A) qRT-PCR analysis for mature miR-101 in five human CRC cell lines and in HCoEpiC cells. U6 was used as an internal reference control. Normalized OGT mRNA levels are presented relative to those in HCoEpiC cells. Values represent the means ± SEM. *** represents Student’s t-test ***P < 0.001, n = 3. Pearson correlation analysis of O-GlcNAc and OGT protein levels with miR-101. **(B)** qRT-PCR analysis of miR-101 in SW-620 cells transfected with a miR-101 mimic vector or a negative control. **(C)** 3D Spheroid BME cell invasion assay of SW-620 cells transfected with a miR-101 mimic vector or negative control. Photographs of all the spheroids in each well every 24 hours for 7 days using a 4× objective. Quantitative analysis of the surface area of all spheroids. Normalized areas for all the spheroids are presented relative to the area on the first day. All the areas were calculated by ImageJ software three times, and the values represent the means ± SEM

Supplemental Figure 5: (A, B, C) Mature miR-101 (a), premiR-101-1 (b) and premiR-101-2 (c) expression levels were assessed by real-time PCR analysis in SW-480 cells after 12 hours of treatment with TMG (10 μmol/L) or isometric DMSO. **(D)** Mature miR-101, premiR-101-1 and premiR-101-2 expression levels in SW-480 cells at 3 days after transfection with 100 nM miR-NC or miR-101.

Supplemental Figure 6: miR-101 is epigenetically silenced by OGT and EZH2 in SW620. (**A, B, C**) ChIP-qPCR assays revealed that H3K27me3, O-GlcNAcylation and EZH2 bind the TSSs of precursor miR-101-1 and miR-101-2 in SW620 cells. (**D**) ChIP-qPCR analysis of O-GlcNAcylation at miR-101's promoter regions in SW620 cells that had been treated with siScramble or siEZH2. (**E**) ChIP-qPCR analysis of H3K27me3 at miR-101's promoter regions in SW620 cells that had been treated with siScramble or siOGT. Normalized O-GlcNAcylation and H3K27me3 levels at the promoter regions of miR-101 in the siEZH2 or siOGT group are presented relative to those in the siScramble group. The values shown represent the means ± SEM.

Supplemental Figure 7: Double-negative feedback loop of the miR-101/O-GlcNAcylation/EZH2 axis in vivo. (**A**) qPCR analysis of the expression of miR-101 in 30 fresh CRC and matched adjacent normal tissues. The values shown are expressed as the means ± SEM. **(B, C)** Scatter plots showing the negative linear correlation between the relative protein expression of OGT (B) or EZH2 (C) and the relative mRNA level of miR-101 in 30 fresh CRC tissues. Normalization was calculated by dividing the expression level of OGT, EZH2 or miR-101 in CRC tissues by the corresponding expression level in the matched adjacent normal tissues. **(D)** Enlarged IHC images of EZH2, O-GlcNAcylation and OGT staining from the tissue microarray, which contains 100 CRC tissues and 80 adjacent normal tissues. The EZH2, O-GlcNAcylation and OGT levels were scored with a semiquantitative IHC analysis. *** represents Student's t-test $P < 0.001$. **(E, F)** Representative images of IHC staining of O-GlcNAcylation, OGT and EZH2 levels and hematoxylin-eosin (HE) staining of 100 CRC tissues and 80 adjacent normal tissues. The high (E) and low (F) expression levels of O-GlcNAcylation were semiquantitatively evaluated by the staining intensity (strong, score: 9–12; medium, score: 6–8, weak, score: 0–4). **(G)** Pearson correlation analysis of O-GlcNAcylation, OGT and EZH2 levels in 100 CRC tissues.
