# Supplementary figures and images for: O-GlcNAcylation promotes colorectal cancer metastasis via the miR-101-O-GlcNAc/EZH2 regulatory feedback circuit
Source: Oncogene. 2018 Aug 9;38(3):301–16. doi: 10.1038/s41388-018-0435-5 (PMC6336687; doi:10.1038/s41388-018-0435-5)

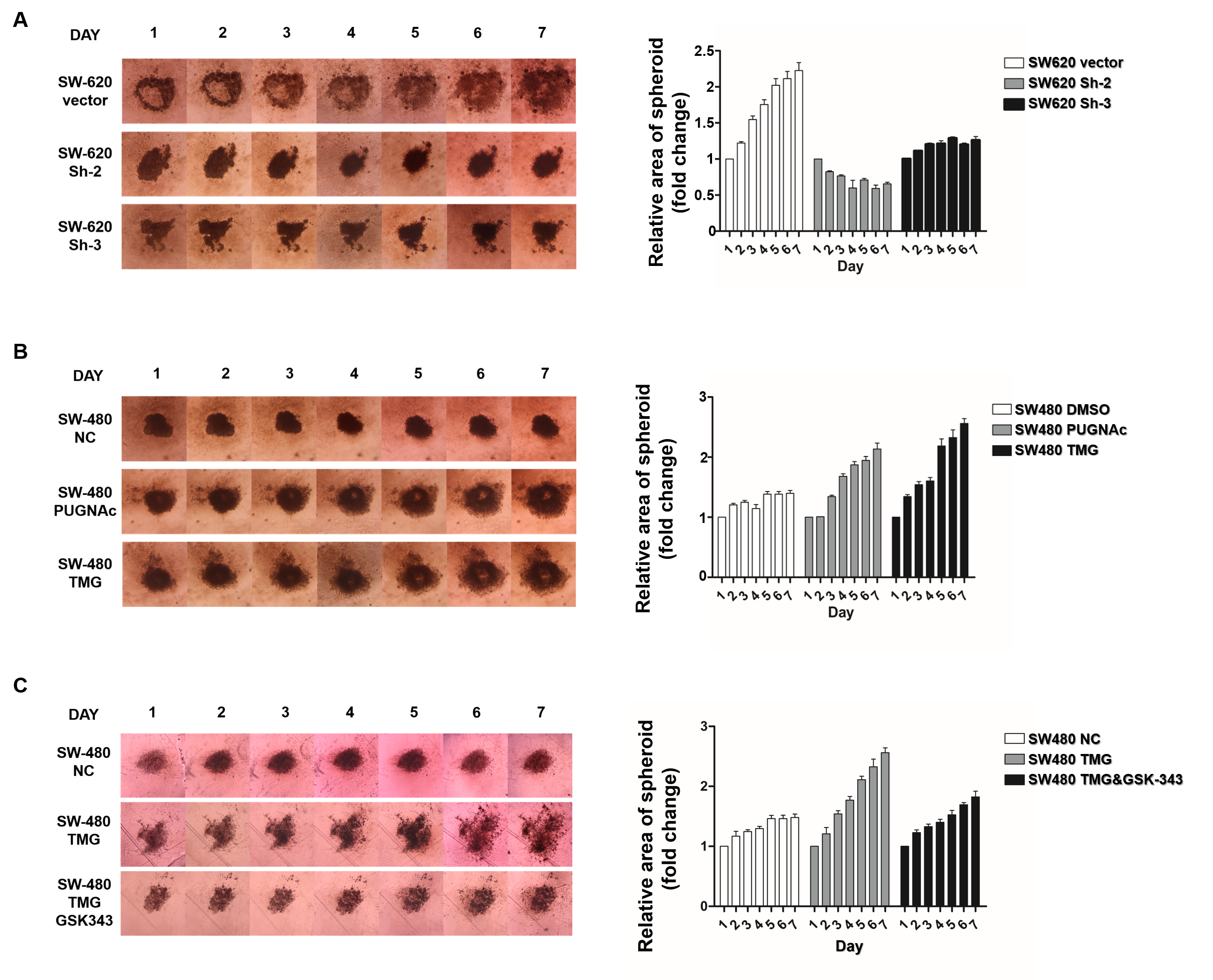

Supplement: Supplementary file 2 — Supplemental figure 1 [file 41388_2018_435_MOESM2_ESM.tif]

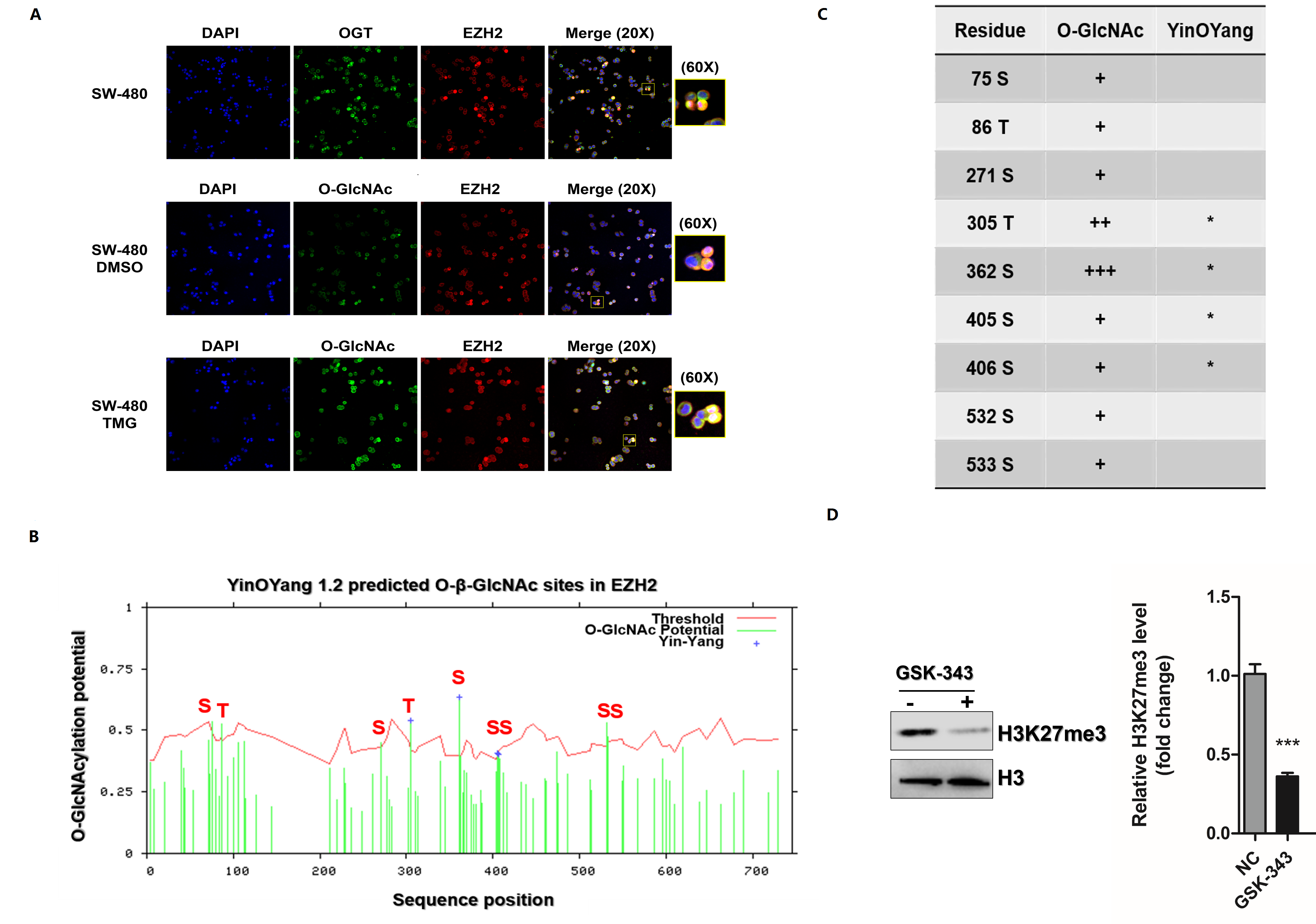

Supplement: Supplementary file 3 — Supplemental figure 2 [file 41388_2018_435_MOESM3_ESM.tif]

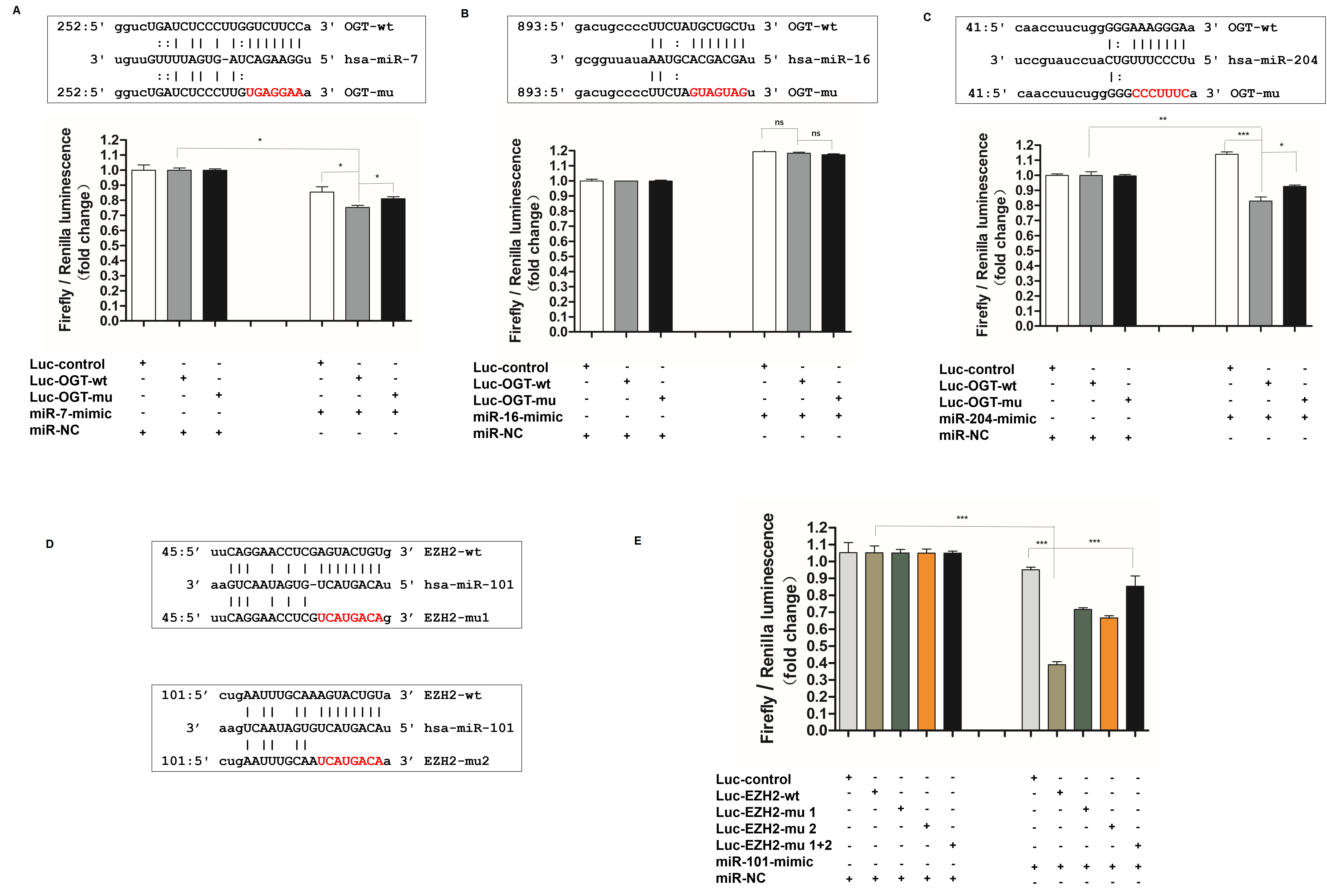

Supplement: Supplementary file 4 — Supplemental figure 3 [file 41388_2018_435_MOESM4_ESM.tif]

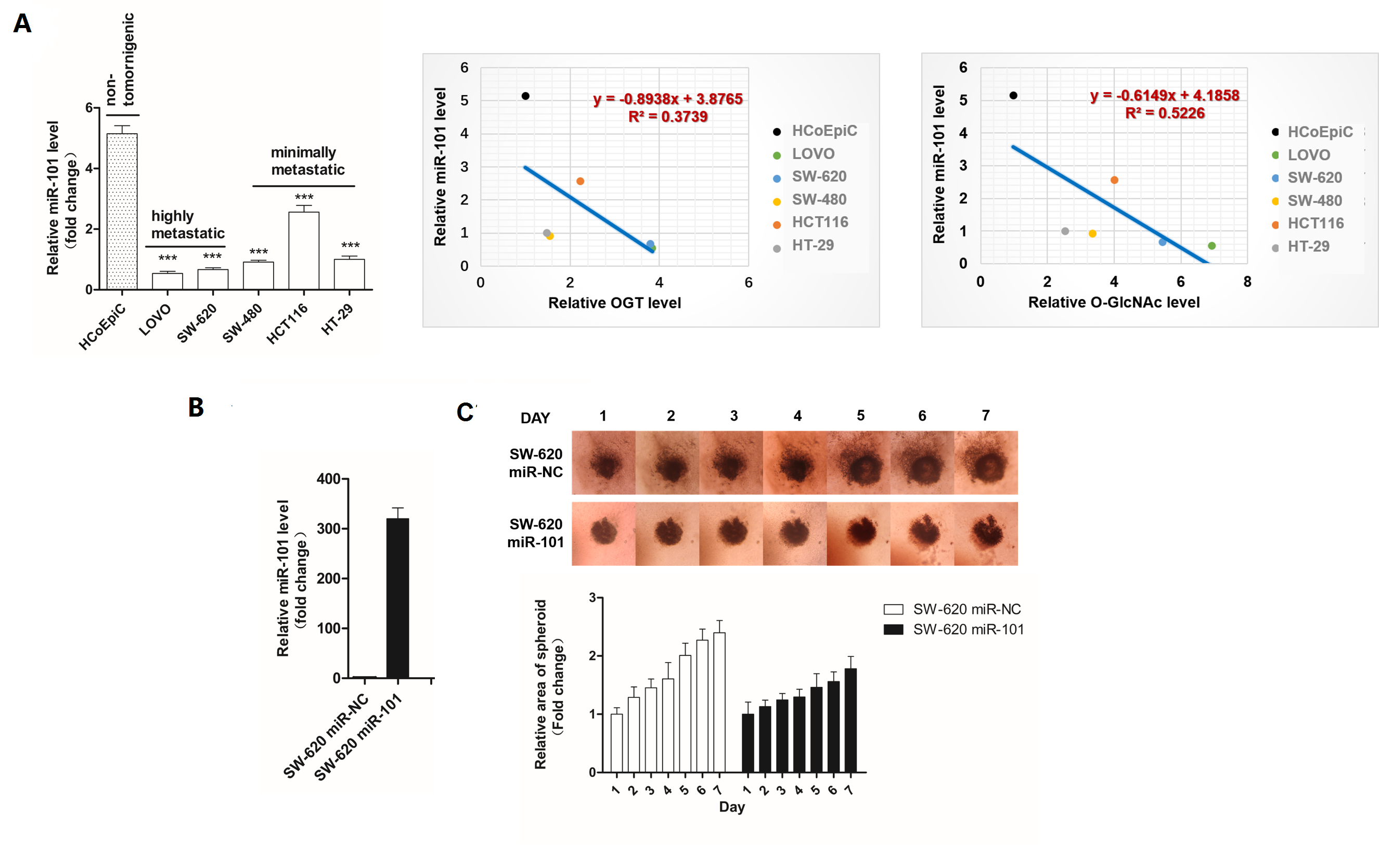

Supplement: Supplementary file 5 — Supplemental figure 4 [file 41388_2018_435_MOESM5_ESM.tif]

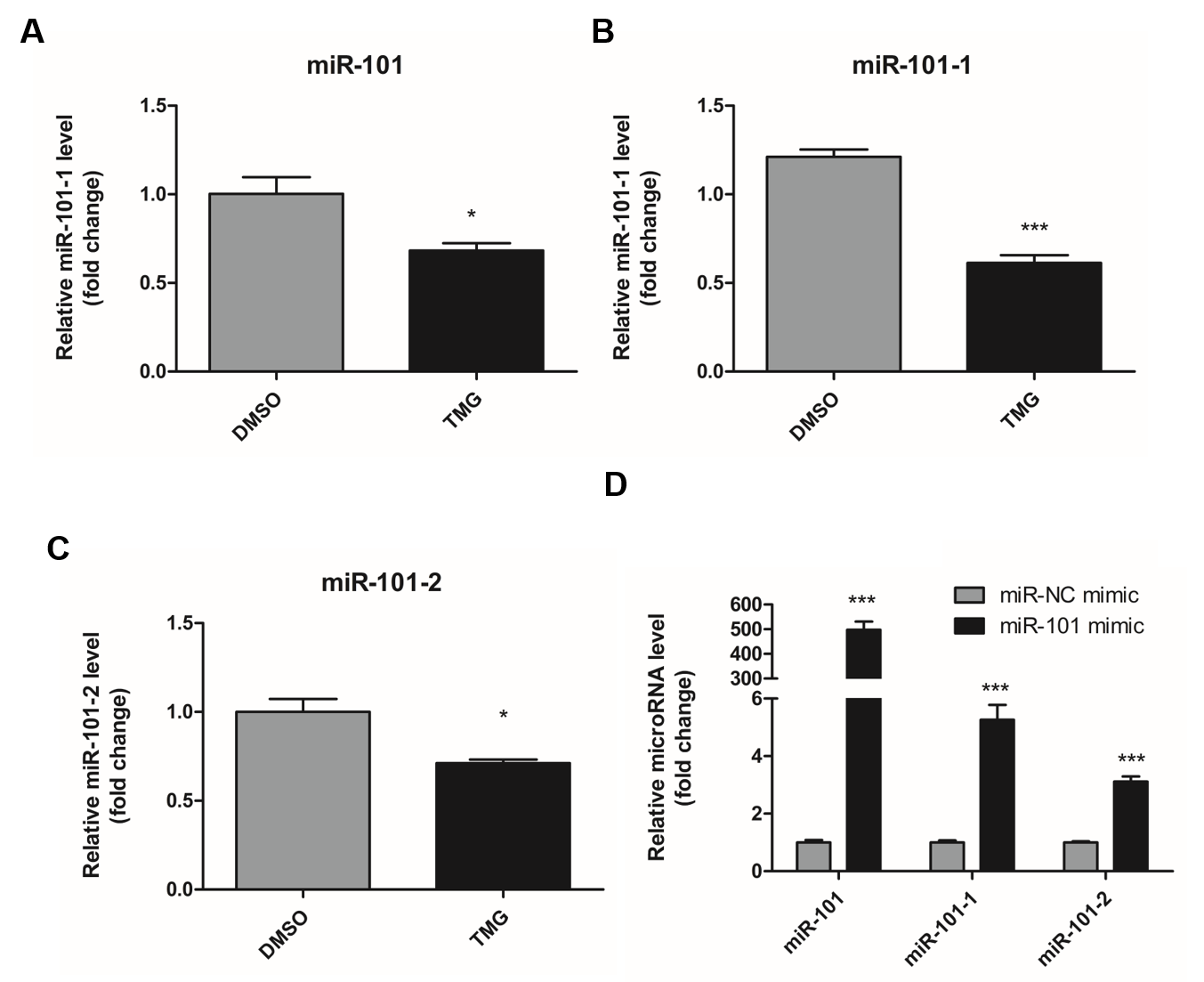

Supplement: Supplementary file 6 — Supplemental figure 5 [file 41388_2018_435_MOESM6_ESM.tif]

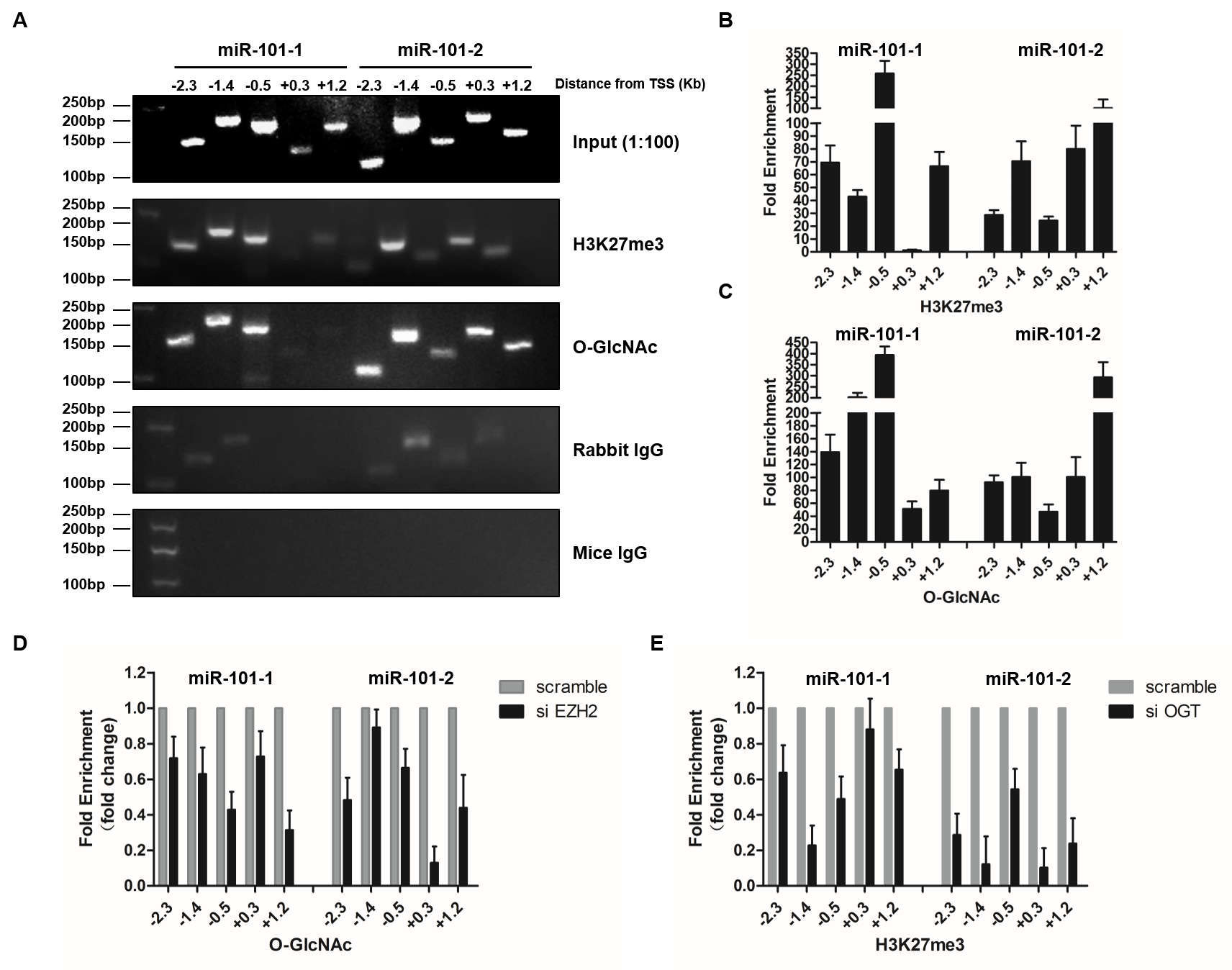

Supplement: Supplementary file 7 — Supplemental figure 6 [file 41388_2018_435_MOESM7_ESM.tif]

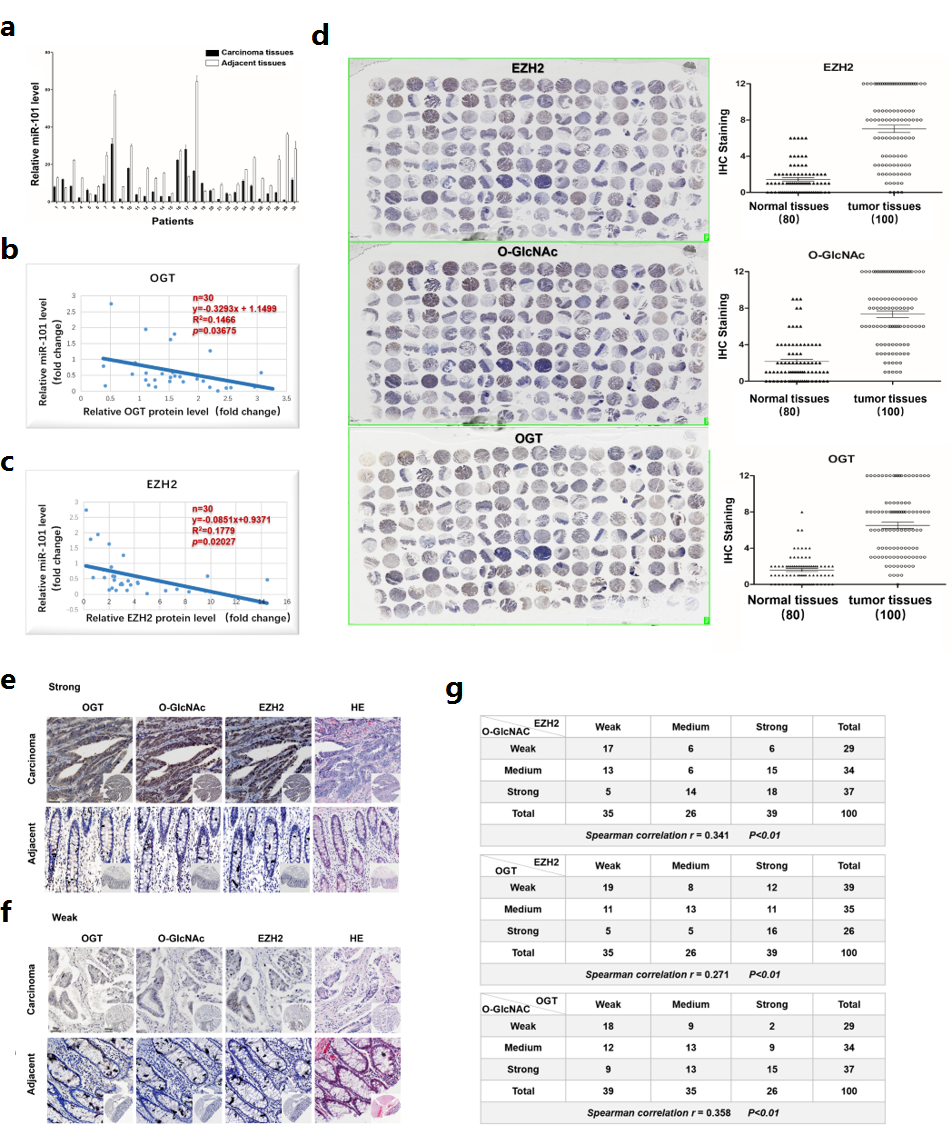

Supplement: Supplementary file 8 — Supplemental figure 7 [file 41388_2018_435_MOESM8_ESM.tif]
